# AVA-NP-695 Selectively Inhibits ENPP1 to Activate STING Pathway and Abrogate Tumor Metastasis in 4T1 Breast Cancer Syngeneic Mouse Model

**DOI:** 10.3390/molecules27196721

**Published:** 2022-10-09

**Authors:** Avijit Goswami, Barnali Deb, Sandeep Goyal, Abhishek Gosavi, Mukund Mali, Ashwita M. Martis, Princy Khurana, Mukesh Gangar, Digambar Raykar, Ankita Mohanty, Aditya Kulkarni

**Affiliations:** 1 AtenPorus Lifesciences, Bangalore 560068, India; 2 Avammune Therapeutics, 39, East Lane, Levittown, PA 19054, USA

**Keywords:** cancer immunotherapy, ENPP1 inhibitor, STING, 2′3′-cGAMP, EMT, 4T1 syngeneic model

## Abstract

Cyclic GMP-AMP synthase (cGAS) is an endogenous DNA sensor that synthesizes cyclic guanosine monophosphate–adenosine monophosphate (2′3′-cGAMP) from ATP and GTP. 2′3′-cGAMP activates the stimulator of interferon genes (STING) pathway, resulting in the production of interferons and pro-inflammatory cytokines. Ectonucleotide pyrophosphatase/phosphodiesterase 1 (ENPP1) is the phosphodiesterase that negatively regulates the STING pathway by hydrolyzing 2′3′-cGAMP. It has been established that the cGAS–STING pathway plays a major role in inhibiting tumor growth by upregulating T cell response. Herein, we demonstrate that AVA-NP-695, a selective and highly potent ENPP1 inhibitor, apart from the immunomodulatory effect also modulates cancer metastasis by negatively regulating epithelial–mesenchymal transition (EMT). We established that the combined addition of 2′3′-cGAMP and AVA-NP-695 significantly abrogated the transforming growth factor beta (TGF-ꞵ)-induced EMT in MDA-MB-231 cells. Finally, results from the in vivo study showed superior tumor growth inhibition and impact on tumor metastasis of AVA-NP-695 compared to Olaparib and PD-1 in a syngeneic 4T1 breast cancer mouse model. The translation of efficacy from in vitro to in vivo 4T1 tumor model provides a strong rationale for the therapeutic potential of AVA-NP-695 against triple-negative breast cancer (TNBC) as an immunomodulatory and anti-metastatic agent.

## 1. Introduction

Cancer is a leading cause of mortality worldwide [1]. One of the breakthroughs in cancer therapy was the discovery of immune checkpoint inhibitors [2]. Despite impressive clinical efficacy shown by anti-cytotoxic T lymphocyte antigen 4 (CTLA-4) and anti-programmed death 1 (PD-1) and its ligand PD-L1 modalities, a large percentage of patients have shown poor response to the treatment [3]. Considering the wide range of limitations shown by the existing immunotherapy regime, there is a constant ongoing effort to decipher ways to boost anti-cancer immunity. One such mechanism involves activation of the cGAS–STING pathway, which plays a crucial role in multiple steps in the cancer immunity cycle [4]. cGAS (cyclic GMP-AMP synthase) is an important cytoplasmic DNA sensor that produces 2′3′-cGAMP, a secondary messenger. 2′3′-cGAMP activates STING, which is located in the endoplasmic reticulum, leading to the recruitment of IRF3 (interferon regulatory factor 3) transcription factor, resulting in the production of type I interferon (IFN-1) and several other pro-inflammatory cytokines [5]. Numerous studies have shown that the activation of STING is important for maintaining anti-tumor responses as it promotes the functional maturation of dendritic cells (DCs), culminating with T cell infiltration and eventually leading to tumor regression [6,7,8]. Recently, several studies have demonstrated the anti-tumor effect of tumor-derived 2′3′-cGAMP [9,10]. These molecular regulations are crucial to boost both the innate and adaptive anti-tumor immune responses [11]. Although several STING agonists are undergoing pre-clinical and clinical trials, the major drawbacks of available STING agonists include poor pharmacokinetic and physicochemical properties, as well as risks of cytokine release syndrome in the case of systemic administration, thereby largely restricting them to intra-tumoral administration [12,13]. Owing to the modest clinical efficacy, the need for an improved modality for activating the pathway for cancer therapy is crucial. One approach to trigger the cGAS–STING pathway is through the inhibition of a downstream enzyme, ENPP1, a negative regulator of the STING pathway which directly hydrolyses 2′3′-cGAMP [14,15]. *ENPP1* is mostly overexpressed in certain tumor cells, e.g., high ENPP1 levels were observed in rat glioma cells, human astrocyte tumors, and also TNBC cells such as 4T1 and MDA-MB-231 [16,17,18,19]. Interestingly, over-expression of *ENPP1* was also reported in M2 macrophages, which play an important role in tumor progression [20,21]. In breast and lung cancer patients, increased *ENPP1* expression was associated with cancer progressing from primary to the metastatic stage [18,19], suggesting an immune escape strategy as it leads to reduced T cell infiltration [18]. ENPP1 not only abolishes the cGAS–STING-mediated immune activation but also produces adenosine, a potent immune suppressor which also promotes cell migration [22]. The metastatic potential of tumor cells is most often acquired through the conserved process of epithelial–mesenchymal transition (EMT) that enhances cell mobility, invasion, and resistance to apoptotic stimuli [23]. Many factors initiate EMT, including the cancer cells in the TME (tumor microenvironment) which secrete several cytokines, most predominant among them being transforming growth factor beta (TGF-β). The molecular pathways classically associated with EMT include TGF-β and Wnt/β-catenin, which are modulated through transcription factors such as Snail/Slug, Twist, Six1, and Cripto [24]. Herein, we have developed an orally available ENPP1 inhibitor, AVA-NP-695, which has demonstrated significantly selectivity towards ENPP1 but does not target other ENPP1 isoforms or several crucial kinases. Although there are other available ENPP1 inhibitors which have shown significant potency, with Ki values ranging from 50 nM to 5 nM across different substrates such as cGAMP or *p*-nitrophenyl 5′-thymidinemonophosphate (*p*-NPh-5′-TMP) [25,26,27,28], our molecule, AVA-NP-695, has shown picomolar Ki (281 pM) using 2′3′-cGAMP as a substrate and 6.25 nM in the case of *p*-NPh-5′-TMP. Furthermore, using AVA-NP-695, we tried to evaluate the molecular mechanisms of EMT reversal mediated by 2′3′-cGAMP and its synergistic potential through ENPP1 inhibition. We also explored the non-inflammatory effect of 2′3′-cGAMP on the reversal of EMT markers induced by TGF-β upon ENPP1 inhibition. Our study not only validates the effect of ENPP1 inhibition in cell lines but also extends its effect in the 4T1 syngeneic model, which showed superior tumor growth inhibition and reduced lung metastasis comparable to existing drugs such as Olaparib and anti-PD1.

## 2. Material and Methods

### 2.1. Cell Culture

The THP1 Dual™ and A549 Dual™ cells were procured from InvivoGen having two inducible reporter constructs, for nuclear factor kappa B (NF-**κ**B) and interferon-stimulated response element (ISRE). THP1 Dual™ cells were maintained in RPMI-1640 (cell clone), 10% FBS (fetal bovine serum) (Gibco), penicillin and streptomycin (100 U/mL) (HiMedia), and the addition of selective antibiotics (Zeocin 100 µg/mL and Blasticidin 10 µg/mL) (InvivoGen). PBMCs were procured from HiMedia (#CL027). MDA-MB-231 (breast cancer) and A549 Dual™ (lung carcinoma) cell lines were cultured in high-glucose (cell clone) DMEM (Dulbecco’s Modified Eagle Medium) with 10% FBS and penicillin and streptomycin (100 U/mL). All cells were maintained at 37 °C and 5% CO_2._

### 2.2. ENPP1 Exogenous Cell-Based Inhibition Assay

ENPP1 exogenous assay was performed using the reported protocol [29]. The final assay reaction mixture contained a buffer of 50 mM Tris pH 9.5 (Sigma-Aldrich, St. Louis, MO, USA), 250 mM NaCl (Sigma-Aldrich), 0.5 mM CaCl_2_ (Sigma-Aldrich), 1 mΜ ZnCl_2_ (Sigma-Aldrich), and 0.1% DMSO (HiMedia). Human recombinant ENPP1 (rENPP1) enzyme (25 ng/well) (R&D Systems) was pre-incubated with 1.25 nM, 12.5 nM, and 125 nM AVA-NP-695 for 15 min at 37 °C in aseptic conditions. The reaction was initiated by adding 100 μΜ 2′3′-cGAMP/well (Invivogen) for A549 Dual™ cells (50 μΜ 2′3′-cGAMP/well for THP1 Dual™) and incubated for 20 min at 37 °C. After incubation, the exogenous reaction mixture was diluted in a 1:12 ratio with media and added to the wells containing 30,000 A549 Dual™ cells or 40,000 THP1 Dual™ cells. IFN induction was determined by QUANTI-Luc™ and luminescence was read using SpectraMax iD3^®®^ (Molecular Devices, San Jose, CA, USA) after 24 h.

### 2.3. QUANTI-Luc™ Luciferase Assay

A549 Dual™ and THP1 Dual™ cell lines were used for performing luciferase-based IFN-β level determination using QUANTI-Luc™. Briefly, 30,000 A549 Dual™ cells or 40,000 THP1 Dual™ cells were seeded and treated with AVA-NP-695 for desired concentrations, followed by treatment of 2′3′-cGAMP after 2 h. QUANTI-Luc™ and cell supernatant was taken in opaque plates after 24 h, and readout was performed using SpectraMax iD3^®®^.

### 2.4. RNA Isolation and Real-Time PCR

Total RNA was isolated from cells using RNA IsoPlus (TaKaRa), and the extracted RNA was quantified for purity using Nanodrop 2000 (Thermo Scientific). The cDNA was prepared using 500 ng of RNA using PrimeScript I strand cDNA Synthesis Kit (TaKaRa). mRNA expression levels of various EMT markers (E-Cadherin (*E-CAD*), N-Cadherin (*N-CAD*), Vimentin (*VIM*), Twist (*TWIST1*)) and several cytokines (*IFN-β*, *TGF-β*, *CXCL-10*, *IL-6*) and *ENPP1* were assessed by real-time PCR (Agilent Technologies) using TB Green^®®^ Premix Ex Taq^™^ II (TaKaRa). All targets were normalized using *GAPDH*, and the fold change was calculated as 2^−ΔΔCt^. Primer sequences are provided in Supplementary Table S3.

### 2.5. Animal Studies

Female BALB/c mice, 6–8 weeks old (Hylasco Biotech (Charles River license, India), were used for determining the efficacy of AVA-NP-695 in vivo. 4T1 Murine Breast cancer cells (ATCC^®®^ CRL-2539™; 0.1 × 10^6^ cells in 1× HBSS) were implanted subcutaneously into the mammary fat pad for developing 4T1 syngeneic tumor models. The tumor-bearing animals were then randomized into 6 different treatment groups of 8 animals per group once the tumor size reached ~50–60 mm^3^. These mice were treated with AVA-NP-695 (1 mg/kg, 3 mg/kg, and 6 mg/kg, BID) and Olaparib (50 mg/kg) orally once daily until the end of the study. Anti-PD1 antibody (10 mg/kg) was administered IP every 3 days. Tumor growth was determined using a digital vernier caliper thrice in a week along with measurements of body weight. The efficacy of the test compound was assessed in terms of tumor growth inhibition (TGI). Percentage tumor growth inhibition (% TGI) between the control and the treated groups was calculated. At the end of the study, lungs were excised and visually observed for metastatic cancer cell colonies. Post-isolation, lungs were fixed in Bouin’s solution and surface lung nodules were counted. All lung specimens were preserved in 10% NBF for further histological evaluation of metastasis.

### 2.6. Lung Histopathology

Formalin-fixed bone samples (tibia and femur) were trimmed for excess muscle and decalcified. Entire lungs and decalcified bone samples were processed according to routine paraffin embedding protocol. This involves dehydration of tissue by increasing grades of alcohol (70%, 95%, and 100%), clearing with xylene, and finally infiltration with paraffin. Following tissue processing, lungs in toto and bone were embedded in molten paraffin. The labeled tissue blocks were sectioned at 4–6 µm thickness using a microtome and sections were flooded over a tissue flotation bath containing water at 45–55 °C. The flattened tissue sections were transferred to a coated slide and allowed to air dry. After drying, slides with the sections were subjected to deparaffinization by using two changes of xylene, followed by rehydration with decreasing grades of alcohol (100%, 95%, and 70%) and finally in water. The sections were subjected to hematoxylin and eosin staining and the excess stain was removed by using running tap water. The tissue sections were then dehydrated and cleared by increasing grades of alcohol (95% and 100%) and xylene. Upon completion of dehydration, stained slides were mounted with DPX mounting medium and subjected to evaluation. To measure the metastatic foci area, 16 random images were captured under 50x magnification and the area was measured in mm^2^ on images using the Zeiss Axioscope A1 microscope equipped with ProgRes^®®^ Capture Pro Software. All the animal experiments were approved by the Institutional Animal Ethics Committee (IAEC) of Anthem Biosciences (CPCSEA registration no. 1192/PO/RcBt/S/08/CPCSEA with approval code ABD/IAEC/PR/195-2020-23 dated 01/06/2020).

### 2.7. KINOMEscan^®®^ Assay

For most assays, kinase-tagged T7 phage strains were grown in parallel in 24-well blocks in an E. coli host derived from the BL21 strain. *E. coli* were grown to log-phase and infected with T7 phage from a frozen stock (multiplicity of infection = 0.4) and incubated with shaking at 32 °C until lysis (90–150 min). The lysates were centrifuged (6000× *g*) and filtered (0.2 µm) to remove cell debris. The remaining kinases were produced in HEK-293 cells and subsequently tagged with DNA for qPCR detection. Streptavidin-coated magnetic beads were treated with biotinylated small molecule ligands for 30 min at room temperature to generate affinity resins for kinase assays. The ligand beads were blocked with excess biotin and washed with blocking buffer (SeaBlock (Pierce), 1% BSA, 0.05% Tween 20, 1 mM DTT) to remove unbound ligand and to reduce non-specific phage binding. Binding reactions were assembled by combining kinases, ligand affinity beads, and test compounds in 1× binding buffer (20% SeaBlock, 0.17× PBS, 0.05% Tween 20, 6 mM DTT). Test compounds were prepared as 40× stocks in 100% DMSO and directly diluted into the assay. All reactions were performed in polypropylene 384-well plates in a final volume of 0.02 mL. The assay plates were incubated at room temperature with shaking for 1 h and the affinity beads were washed with wash buffer (1× PBS, 0.05% Tween 20). The beads were then re-suspended in elution buffer (1× PBS, 0.05% Tween 20, 0.5 µM non-biotinylated affinity ligand) and incubated at room temperature with shaking for 30 min. The kinase concentration in the eluates was measured by qPCR. AVA-NP-695 were screened at the 10 μM, and the results for primary screen binding interactions are reported as %Ctrl, where lower numbers indicate stronger hits in the matrix. Selectivity score or S-score is a quantitative measure of compound selectivity. It is calculated by dividing the number of kinases that compounds bind to by the total number of distinct kinases tested, excluding mutant variants.

S = Number of hits/Number of assays

This value can be calculated using %Ctrl as a potency threshold (below) and provides a quantitative method of describing compound selectivity to facilitate the comparison of different compounds.

S (35) = (number of non-mutant kinases with %Ctrl <35)/(number of non-mutant kinases tested)

S (10) = (number of non-mutant kinases with %Ctrl <10)/(number of non-mutant kinases tested)

S (1) = (number of non-mutant kinases with %Ctrl <1)/(number of non-mutant kinases tested)

### 2.8. Bioinformatic Analysis

Publicly available sequencing data were analyzed using the cBioPortal for Cancer Genomics and the UALCAN analysis tool [30,31,32]. mRNA and protein expression were determined using TCGA (The Cancer Genome Atlas) and CPTAC (Clinical Proteomic Tumor Analysis Consortium) datasets, respectively. For analyzing the expression levels across cancer types, TCGA Pan-Cancer dataset was selected in UALCAN and a bar graph was plotted, showing the gene expression levels of ENPP1 across cancers. ENPP1 protein and gene expression correlation was conducted using cBioPortal. The CCLE datasets were considered for the analysis. A correlation matrix was plotted for all the cell lines, where MDA-MB-231 and A549 cells were highlighted for better representation. All the figures and generated plots were edited using Adobe Illustrator 2021 (vCS5.1; Adobe Systems, San Jose, CA, USA).

### 2.9. Statistical Analysis

Experiments were carried out in triplicates and plotted using GraphPad Prism. Comparisons were performed using Student’s *t*-test with two-tailed distribution at a confidence level of 95%. All error bars indicate SD. Comparisons between multiple groups were performed using ordinary one-way analysis of variance (ANOVA) followed by Dunnett’s test. For normality, Welch’s correction was used and Bartlett’s test was used for homogeneity of variance tests. All error bars indicate SD. Test results are reported as *p*-values with a significance cutoff set at *p* < 0.05. All analyses were performed using GraphPad 9.0 software (GraphPad Software Inc.).

## 3. Results

### 3.1. Differential Expression of ENPP1 across Cancer Types

**Figure 1:**
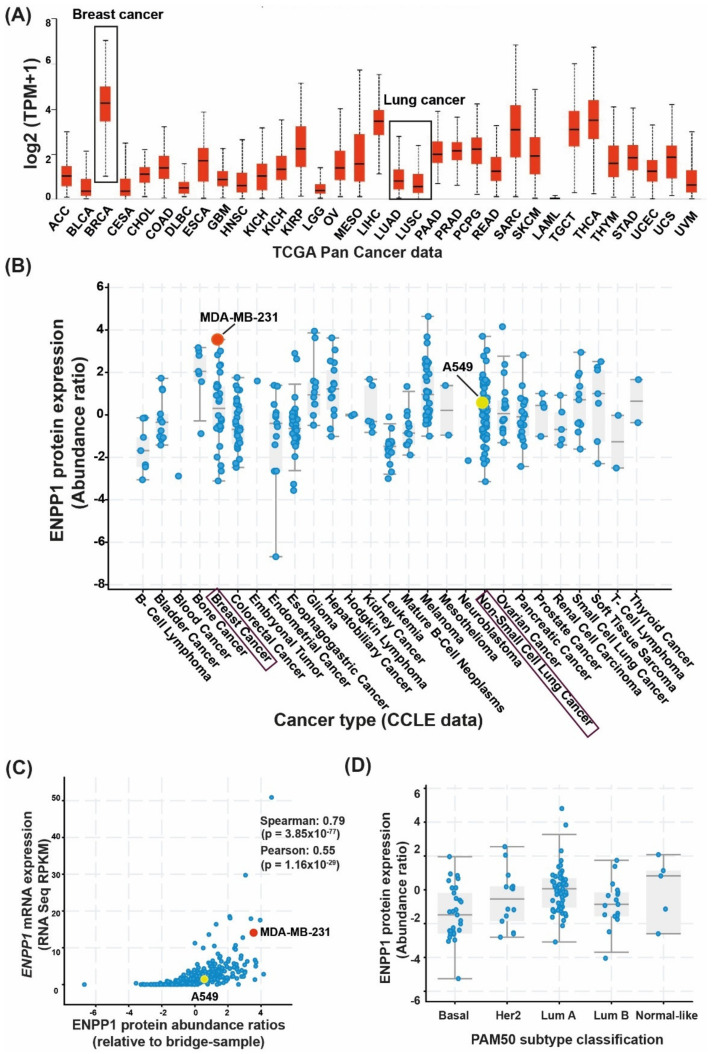
ENPP1 expression across various cancer types. Publicly available sequencing datasets were analyzed to determine the gene and protein expression of ENPP1 across cancer types. (**A**) The Cancer Genome Atlas (TCGA) RNA sequencing data from tumor tissue samples were analyzed using the UALCAN analysis tool. The gene expression of *ENPP1* across cancer types has been shown, where breast cancer shows the highest expression (4.27 log2(TPM+1)). (**B**) Protein abundance of ENPP1 across cancer cell lines. The data were acquired from the CCLE database and analyzed through cBioPortal. MDA-MB-231 (triple-negative breast cancer cell line) shows an abundance ratio of 3.55 (ratio with adjacent normal). A549 (lung adenocarcinoma) had an abundance ratio of 0.58. (**C**) Pearson’s correlation analysis showed the correlation between mRNA and protein expressions of ENPP1. The mRNA expression levels are positively correlated with the protein expression across all cancer cell lines (r = 0.55; *p* = 1.16 × 10^−29^). MDA-MB-231 and A549 cell lines are highlighted in red and yellow, respectively. (**D**) The PAM50-classified breast cancer samples [35] have been analyzed to depict the levels of ENPP1 protein abundance across the molecular subtypes.

The gene and protein expression levels of ENPP1 have been reported to be upregulated in several cancer types [33,34]. However, to confirm the levels of ENPP1, pan-cancer RNA and protein sequencing datasets from primary tumor tissue samples as well as cancer cell lines (CCLE datasets) were analyzed using cBioPortal and UALCAN [30] analysis tools. At first, the *ENPP1* expression was analyzed using The Cancer Genome Atlas Program (TCGA) pan-cancer datasets. *ENPP1* expression was considerably high in breast cancer samples (median value: 4.27 log2(TPM+1)) and significantly low in lung cancer patients (median value: 0.8 and 0.5 log2(TPM+1) in lung adenocarcinoma and lung squamous cell carcinoma, respectively) (Figure 1A). *ENPP1* expression levels across cancer cell lines (CCLE datasets) were also analyzed to find the concordance of *ENPP1* expression between patient samples and cell lines. ENPP1 was found to be overexpressed in MDA-MB-231 (triple-negative breast cancer) with an abundance ratio of 3.55 (ratio with adjacent normal) as compared to A549 (lung adenocarcinoma), which had an abundance ratio of 0.58 (Figure 1B). Thus, MDA-MB-231 cells were considered as “high-ENPP1” and A549 as “low-ENPP1” for subsequent experiments. Additionally, the gene and protein expression levels of ENPP1 from the CCLE RNA sequencing and global proteomic datasets were significantly correlated (*r* = 0.55; *p* = 1.16 × 10^−29^) (Figure 1C). The expression of *ENPP1* across PAM50 subtypes (molecular classification) (Figure 1D) as well as tumor stages (Figure S1) did not vary significantly.

Cumulatively, these data suggest that ENPP1 has high gene and protein expression levels across cancer types, especially in breast cancer. The expression levels of *ENPP1* are lower in lung cancer. Moreover, the gene and protein expression levels are positively correlated across cancers.

### 3.2. In Vitro Efficacy of AVA-NP-695 as a Potent Small ENPP1 Inhibitor

**Figure 2:**
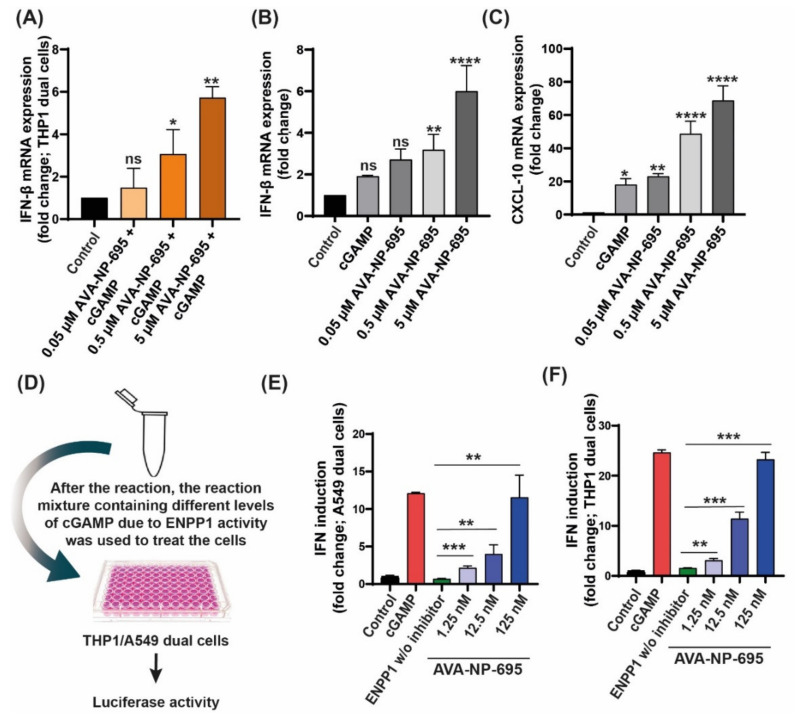
AVA-NP-695 significantly induces IFN response in various in vitro cell studies. (**A**) Effect of AVA-NP-695 on *IFN-β* mRNA level. THP1 Dual^TM^ cells were treated with different AVA-NP-695 concentrations and 25 µM of 2′3′-cGAMP for 24 h and RNA was isolated. *IFN-β* fold change was plotted; data were normalized using *GAPDH*. AVA-NP-695 was added 2 h prior to 2′3′-cGAMP addition (n = 3). (**B**,**C**) Three million PBMCs were seeded in a 12-well plate. AVA-NP-695 was added to the respective wells at various concentrations. The plate was incubated for 2 h prior to addition of 2′3′-cGAMP (25 µM). The plate was further incubated for 24 h. RNA was isolated after the incubation, followed by RT-PCR for *IFN-β* (**B**) and *CXCL-10* (**C**) mRNA quantification. *GAPDH* was used as endogenous control (n = 2). (**D**) Schematic representation of functional effect of ENPP1 inhibition on IFN induction in (**E**) A549 Dual^TM^ and (**F**) THP1 Dual^TM^ cells. After the in vitro enzymatic reaction, 10 µL of the reaction mixture was diluted by adding 120 µL of complete RPMI. This diluted mix was used to treat the cells for 24 h. IFN induction was estimated by a luciferase reporter assay under the control of IFN-stimulated response elements. For all the experiments, control groups were considered to be 1 and the *p*-values were calculated with respect to the control group using ordinary one-way analysis of variance followed by Dunnett’s test. For comparison between two internal groups, Dunnett’s multiple comparison test was performed. * p < 0.05, ** *p* < 0.01, *** *p* < 0.001, **** *p* < 0.0001, ns: non-significant.

We developed a potent in-house ENPP1 inhibitor, AVA-NP-695, to study the effects of ENPP1 inhibition on various therapeutic aspects of cancer. It is known that ENPP1 is a member of the conserved ectonucleotide pyrophosphatase/phosphodiesterase (ENPP) family comprising various other isoforms [36]. It was observed that AVA-NP-695 selectively inhibits ENPP1 and no other ENPP isoforms (ENPP2, ENPP3, ENPP5, ENPP6, and ENPP7) at a 100 µM concentration (Figure S2A). The IC_50_ of 14 ± 2 nM was noted through an enzymatic assay using *p*-Nph-5′-TMP substrate to check the inhibitory potency of AVA-NP-695 (Figure S2B). To demonstrate the effect of ENPP1 inhibition on activation of the STING pathway and the downstream cytokine production, THP1 Dual™ cells were treated with AVA-NP-695 (0.05 μM, 0.5 μM, and 5 μM) in combination with 2′3′-cGAMP (25 μM), and the *IFN-β* mRNA level and IFN response using QUANTI-Luc™ luciferase assay were measured (Figure 2A, Figure S2C). The 2′3′-cGAMP concentration for the treatment was finalized based on a dose-dependent IFN induction on THP1 Dual™ cells (Figure S3). Finally, the selectivity of AVA-NP-695 across 469 kinases was tested to understand the off-target effect using KINOME*scan*^®®^ (Figure S2D, Supplementary Table S1). Out of 403 non-mutant kinases tested for binding with AVA-NP-695 at 10 μM, no binding was observed in 446 kinases, %Ctrl <35 was observed for 18 kinases, %Ctrl <10 was observed for 4 kinases, and %Ctrl <1 was observed for 1 kinase (Supplementary Table S1). Overall, these data confirm the high selectivity of AVA-NP-695 towards ENPP1 and the reduced chances of off-target effects across the diverse kinase panel.

A dose-dependent increase in IFN response was confirmed by evaluating the *IFN-β* and *CXCL10* mRNA expression in human peripheral blood mononuclear cells (PBMCs) (Figure 2B,C). Despite the low IC_50_ of the inhibitor (14 nM) in an enzymatic assay, we observed a significant increase in IFN induction only at higher doses (0.5 μΜ and 5 µM). This could be attributed to the low normalized expression of ENPP1 in THP1 Dual™ cells. Hence, we evaluated the compound in an in vitro exogenous ENPP1 assay that has been previously reported [29]. The dose dependency of AVA-NP-695 significantly reflected the IFN induction at a low concentration of 12.5 nM. The fold induction almost matches that of the 2′3′-cGAMP control at the highest concentration of 125 nM, suggesting complete inhibition of ENPP1 and enhanced stability of 2′3′-cGAMP leading to significantly high IFN induction (Figure 2D–F). These data validate the efficacy of AVA-NP-695 towards selectively targeting ENPP1 and also suggest that the cellular potency of the inhibitor largely depends on the target expression level in the cell type used for in vitro studies.

### 3.3. 2′3′-cGAMP Modulates EMT Markers Based on the Cellular ENPP1 Level

Recently, 2′3′-cGAMP has been reported to suppress EMT in cancer cells [37]. As ENPP1 levels modulate 2′3′-cGAMP levels and therefore affect its anti-tumor activity, we speculated the involvement of ENPP1 in EMT modulation as well. Initially, the *ENPP1* expression levels in MDA-MB-231 and A549 Dual™ were estimated.

**Figure 3:**
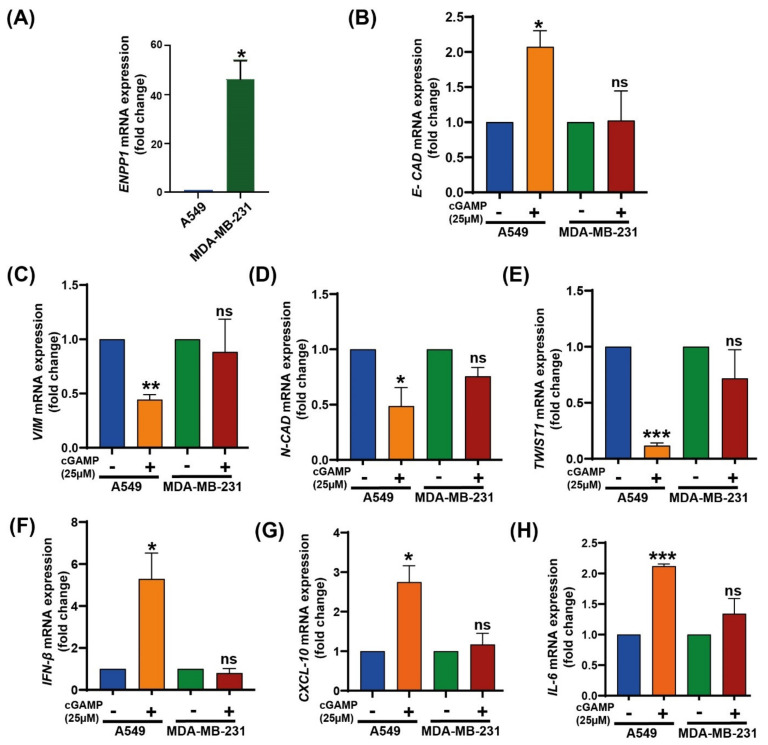
Effect of 2′3′-cGAMP on EMT markers. (**A**) *ENPP1* mRNA levels were quantified in A549 Dual™ and MDA-MB-231. Ct values of *ENPP1* were normalized with respective Ct values for *GAPDH* (normalized Ct). Relative fold change was plotted considering A549 Dual™ as 1 (n = 3). (**B**–**E**) A549 Dual™ and MDA-MB-231 cells were treated with 25 µM 2′3′-cGAMP for 24 h, and mRNA levels of various EMT markers were estimated using RT-PCR (**B**) *E-CAD*, (**C**) *VIM*, (**D**) *N-CAD*, and (**E**) *TWIST1* (n = 3). (**F**–**H**) Inflammatory cytokine mRNA levels were also quantified in the same experiment (**F**), *IFN-β,* (**G**), *CXCL-10*, (**H**), and *IL-6*. The fold change values were plotted using *GAPDH* as endogenous control (n=3). The *p*-values were calculated using paired Student’s *t*-test. * *p* < 0.05, ** *p* < 0.01, *** *p* < 0.001, ns: non-significant.

It was observed that *ENPP1* expression was significantly higher in MDA-MB-231 (~50 fold) than in A549 Dual™ cells (*p =* 0.0097) (Figure 3A), consistent with the bioinformatics analysis. To further evaluate the alterations in EMT markers in the presence of 2′3′-cGAMP, A549 and MDA-MB-231 cells were treated with 2′3′-cGAMP (25 µM). It was found that the expression of *E-CAD* (*p* = 0.0149) was significantly upregulated with 2′3′-cGAMP treatment, whereas *VIM* (*p* = 0.0011), *N-CAD* (*p* = 0.033) and *TWIST1* (*p* = 0.0003) were significantly downregulated in A549, i.e., low-ENPP1 cells (Figure 3B–E). On the contrary, there were no significant changes in the expression of EMT markers or cytokines in the high-ENPP1 cells (Figure 3B–E). Expectedly, *IFN-β* (*p* = 0.026), *CXCL-10* (*p* = 0.018) and *IL-6* (*p* = 0.0004) expression levels were found to be elevated in 2′3′-cGAMP-treated A549 Dual™ cells, along with no significant difference in MDA-MB-231 cells (Figure 3F–H).

### 3.4. ENPP1 Determines 2′3′-cGAMP-Mediated Modulation of EMT Markers

**Figure 4:**
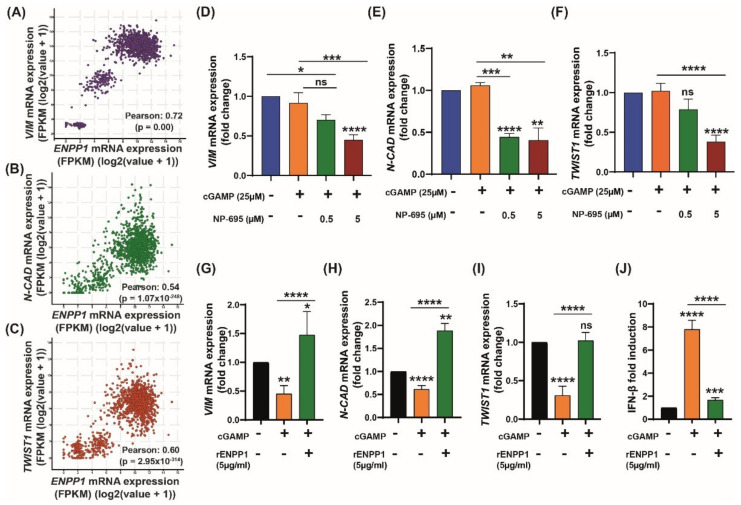
ENPP1 facilitates EMT by modulating 2′3′-cGAMP levels in MDA-MB-231 and A549 Dual™ cells. (**A–C**) Pearson’s’ correlation was conducted on TCGA breast cancer data using cBioPortal. The mRNA expressions of mesenchymal markers (**A**) *VIM*, (**B**) *N-CAD,* and (**C**) *TWIST1* were analyzed for their correlation with *ENPP1* expression (r > 0.5, *p* < 0.05). (**D–F**) Effect of ENPP1 inhibitor (5 µM) on EMT markers at mRNA level with 2′3′-cGAMP (25 µM) in high-ENPP1 expression MDA-MB-231 cells. (**D**) *VIM,* (**E**) *N-CAD,* and (**F**) *TWIST* expression was evaluated through real-time PCR and data were normalized using *GAPDH* (n = 3). (**G**–**J**) EMT markers elevated in the presence of rENPP1 in A549 Dual™ cells. rENPP1 (5 µg/mL) along with 2′3′-cGAMP (25 µM) was added to A549 Dual™ cells and incubated for 24 h. mRNA levels were evaluated using RT-PCR. (**G**) *VIM*, (**H**) *N-CAD*, (**I**) *TWIST1*, and (**J**) *IFN-β* (n = 3). The *p*-values were calculated with respect to the no-treatment group using ordinary one-way analysis of variance followed by Dunnett’s test. For comparison between two internal groups, Dunnett’s multiple comparison test was performed. * *p* < 0.05, ** *p* < 0.01, *** *p* < 0.001, **** *p* < 0.0001, ns: non-significant.

Next, we checked the correlation of EMT markers with high expression of *ENPP1.* It was observed that in TCGA breast cancer datasets, the high expression of *ENPP1* was significantly correlated (Pearson’s correlation) with the expression of *VIM* (*r* = 0.72; *p =* 0.00), *N-CAD* (*r* = 0.54; *p* = 1.07 × 10^−248^), and *TWIST1* (*r* = 0.60; *p* = 2.95 × 10^−314^) (Figure 4A–C). Furthermore, the effect of ENPP1 inhibition on the modulation of EMT markers through 2′3′-cGAMP was investigated. For this study, MDA-MB-231 cells were selected, which exhibited high-ENPP1 expression for efficient inhibition. The 2′3′-cGAMP-treated MDA-MB-231 cells were treated in the presence and absence of AVA-NP-695 (0.5 and 5 µM). It was observed that simultaneous treatment of the cells with 2′3′-cGAMP and AVA-NP-695 significantly reduced the expression of the mesenchymal markers, namely *VIM* (*p* = 0.0155) and *N-CAD* (*p* < 0.0001) (Figure 4D,E). Additionally, these observations were enhanced with the increase in concentration (5 µM), where *TWIST1* (*p* < 0.0001) was downregulated (Figure 4F) and *E-CAD* (*p* = 0.0023) was found to be upregulated (Figure S4A). As anticipated, the expression of *IFN-ꞵ* was also significantly upregulated (*p* = 0.016) (Figure S4B).

To establish that the effect of 2′3′-cGAMP on EMT modulation was mediated by the STING pathway, the A549 Dual™ cells were treated with H-151 [38] (a selective STING inhibitor; 0.5 and 5 µM) in the presence of 2′3′-cGAMP. Upon STING inhibition, there was a significant decrease in *E-CAD* expression, as well as type 1 interferon (*IFN-ꞵ* and *CXCL10*) expression. Subsequently, the decrease in *VIM* level observed in the case of cGAMP treatment was rescued back to normal (Figure S5A–D). The involvement of the STING pathway was further confirmed in a follow-up experiment using HT-DNA as an activator of cGAS–STING. A significant decrease in *VIM* and *N-CAD* mRNA levels was observed upon treatment with HT-DNA and showed complete rescue in the presence of H151 (Figure S5E–F). This confirms a direct role of the STING pathway in modulating the EMT. To validate the role of ENPP1 in EMT regulation via 2′3′-cGAMP, low-ENPP1-expressing A549 Dual™ cells were replenished with recombinant human ENPP1 (rENPP1) and the EMT markers were evaluated. Interestingly, the effect of 2′3′-cGAMP on EMT levels in A549 Dual™ cells was significantly reversed in the presence of rENPP1 (Figure 4G–I). Since these A549 Dual™ cells expressed an IFN response element, 2′3′-cGAMP-mediated IFN induction could be directly quantified using a luciferase assay. rENPP1 significantly reduced the IFN induction, which indicated 2′3′-cGAMP hydrolysis (Figure 4J). Overall, these results suggest that 2′3′-cGAMP is able to modulate EMT only in low-ENPP1 cells. However, solitary induction of 2′3′-cGAMP is not sufficient in high-ENPP1 cells to modulate EMT. Only a combination of 2′3′-cGAMP and ENPP1 inhibitor—in this case, AVA-NP-695—is able to restore the EMT levels, thus highlighting the importance of ENPP1 inhibitors in high-ENPP1 cancer (Figure 4G–J).

### 3.5. 2′3′-cGAMP Blocks EMT Induced by TGF-β in Presence of ENPP1 Inhibitor, AVA-NP-695

**Figure 5:**
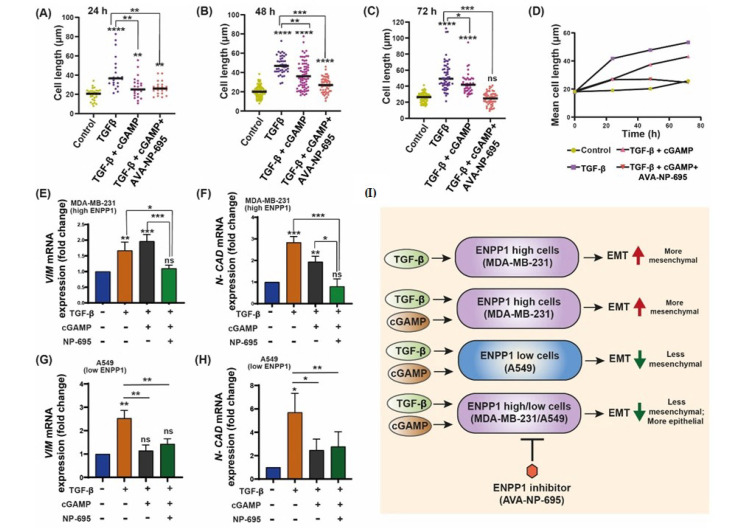
The effect of 2′3′-cGAMP and TGF-ꞵ in the presence of ENPP1 inhibitor on the expression level of EMT markers. (**A**–**C**) EMT was induced in MDA-MB-231 cells through TGF-ꞵ treatment (10 ng/mL) for periods of 24, 48, and 72 h. The cells were consecutively treated with 2′3′-cGAMP alone and in combination with AVA-NP-695. (**D**) Mean cell lengths with the treatment of TGF-ꞵ along with 2′3′-cGAMP and AVA-NP-695 from each time point and group were plotted. Cell length was analyzed using ImageJ. (**E**,**F**) The gene expression of *VIM* and *N-CAD* was evaluated through real-time PCR and the data were normalized using *GAPDH* (n = 3). (**G**,**H**) Low-ENPP1-expressing A549 Dual™ cells were treated with TGF-β, cGAMP, and TGF-β and TGF-β, cGAMP, and AVA-NP-695 for 48 h; *VIM* (**G**) and *N-CAD* (**H**) mRNA expression levels were evaluated using RT-PCR (n = 3). (**I**) The schematic representation showing the cumulative conclusion of the experiment. The *p*-values were calculated with respect to the no-treatment group using ordinary one-way analysis of variance followed by Dunnett’s test. For comparison between two internal groups, Dunnett’s multiple comparison test was performed. *<0.05, ** *p* < 0.01, *** *p* < 0.001, **** *p* < 0.0001.

Metastasis stimulates the spread of cancer, responsible for up to 90% of patient deaths, which is largely caused by EMT [39]. Numerous factors initiate EMT, including the cancer cells in the TME (tumor microenvironment), which secrete many cytokines, most predominant among them being TGF-β [40]. TGF-ꞵ, a multi-functional cytokine in mammals, plays a crucial role in the alteration of the cytoskeleton [41]. It has been reported that TGF-ꞵ induces tumor formation by promoting EMT [42], cell elongation [43], movement in vitro, and cancer metastasis in vivo [44]. In a recent study, MDA-MB-231 cells were treated with TGF-ꞵ (10 ng/mL) for 48 h and transcriptomic analysis was carried out [45]. After establishing the EMT-modulating mechanism of 2′3′-cGAMP, we hypothesized that similar results could be achieved when EMT is induced in high-ENPP1 MDA-MB-231 cells. Consequently, we induced EMT in MDA-MB-231 cells through TGF-ꞵ treatment (10 ng/mL) for a period of 72 h. TGF-ꞵ-induced EMT was confirmed by upregulated *VIM* and *TWIST1* mRNA and downregulated *E-CAD* mRNA levels (Figure S6). Interestingly, the EMT traits displayed by the cells after treatment with TGF-ꞵ were significantly abrogated upon 2′3′-cGAMP and AVA-NP-695 treatment. As early as 24 h after treatment of the TGF-ꞵ, the cell length was significantly increased (*p* < 0.0001). However, with the treatment of 2′3′-cGAMP (after TGF-ꞵ induction), the cells showed moderately less cell length, which further decreased with the treatment of AVA-NP-695 in combination with 2′3′-cGAMP (Figure 5A and Figure S7). Similar results were observed after 48 h and 72 h treatment (Figure 5B,C, Figure S7). Thus, a decrease in mean cell length with the treatment of TGF-ꞵ along with 2′3′-cGAMP and AVA-NP-695 was observed in MDA-MB-231 cells treated for 24 h, 48 h, and 72 h (Figure 5D, Supplementary Table S2). 2′3′-cGAMP-treated cells also showed sufficient loss in cell length in the TGF-ꞵ-induced cells, although to a lesser extent as compared to the combination of both 2′3′-cGAMP and AVA-NP-695. Interestingly, only the 2′3′-cGAMP+ AVA-NP-695 group depicted a significant decrease in *VIM* (*p* = 0.006) and *N_CAD* (*p* < 0.0001) mRNA levels compared to the group treated with TGF-ꞵ alone or the group treated with TGF-ꞵ and 2′3′-cGAMP (Figure 5E,F).

Overall, this observation is consistent with a previous report which demonstrated that ENPP1 knockdown suppressed clonogenic formation and tumorigenicity in vivo [34]. Next, to further confirm that the level of ENPP1 is crucial in modulating EMT by cGAMP, we performed a similar experiment in low-ENPP1-expressing A549 Dual™ cells. We observed that cGAMP significantly abrogated TGF-β-induced upregulation of *VIM* (*p* < 0.0034) and *N-CAD* (0.0006), and the addition of AVA-NP-695 caused a similar effect to that of cGAMP treatment compared to treatment with TGF-β alone (Figure 5G–H). These results suggest that in a given context where TGF-β and 2′3′-cGAMP are both present, the influence of TGF-β surpasses that of 2′3′-cGAMP, primarily due to constant hydrolysis of 2′3′-cGAMP by ENPP1, provided that the ENPP1 expression is high. These observations indicate that in a high-ENPP1-expressing condition, 2′3′-cGAMP treatment alone is insufficient to reverse EMT in TGF-ꞵ-treated cells. However, once the ENPP1 is inhibited by AVA-NP-695, or there re low-ENPP1-expressing cells, the impact of cGAMP is pronounced to reverse EMT (Figure 5I).

### 3.6. Pharmacological Inhibition of ENPP1 by AVA-NP-695 Abrogates 4T1 Tumor Growth and Metastasis in Syngeneic Mice Model

**Figure 6:**
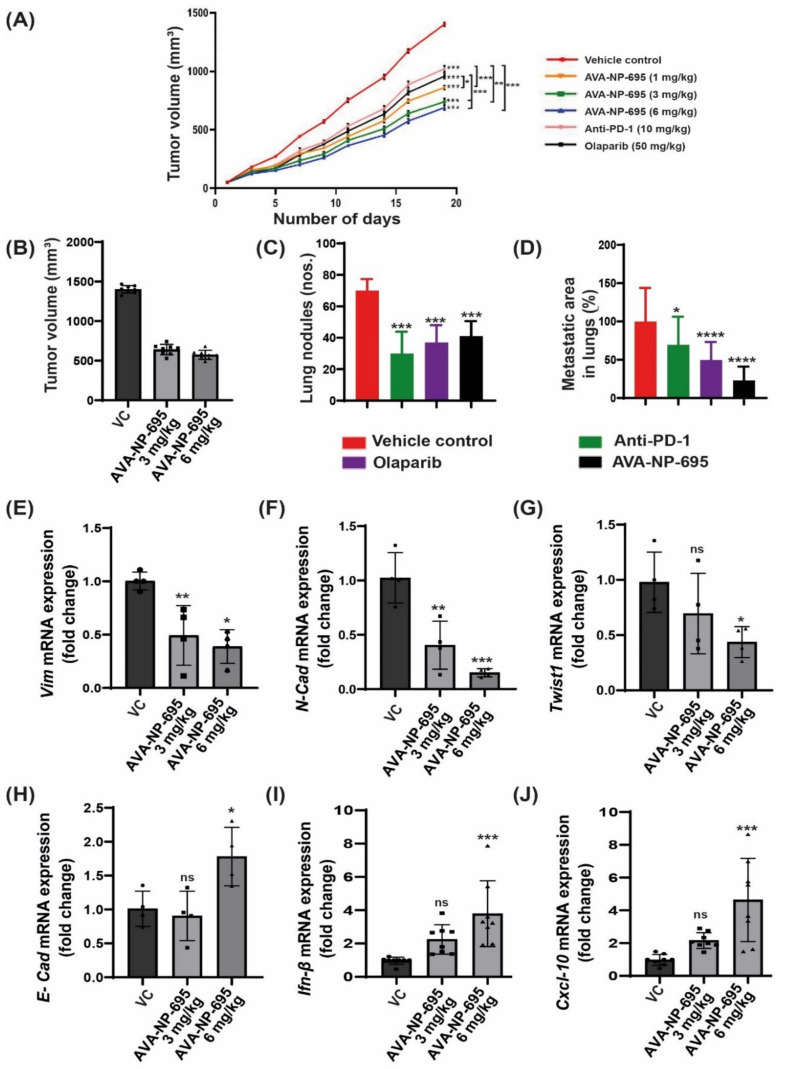
AVA-NP-695 demonstrates superior anti-tumor efficacy and reduces metastasis in the 4T1 syngeneic model. (**A**) Tumor volume at given time points was measured with different concentrations of AVA-NP-695 (1 mg/kg, 3 mg/kg, 6 mg/kg) and anti-PD1 and Olaparib and the growth kinetics of the mice after treatment (n = 8). All the animals were sacrificed on day 19. (**B**) Tumor volume at day 19 was plotted for the vehicle and 2 dose of AVA-NP-695 (3 and 6 mg/kg) (n = 8). (**C**) The metastatic lung nodules by 4T1 cells were visually counted for each animal of the respective group indicated in the graph and the average number of nodules is presented as mean ± SD (n = 8) after sacrificing the animals on day 19. (**D**) Histological analysis of lung metastasis of 4T1 tumors from various treatment groups were stained with hematoxylin and eosin. The percentage of metastasized area was calculated by considering the mean metastatic area of the vehicle group as 100%: [(metastatic area/mean metastatic area of vehicle) × 100)] (n = 8). (**E**–**J**) The mRNA expression levels of EMT markers and cytokines were quantified through real-time PCR in the animal tissue samples treated with AVA-NP-695 at 3 mg/kg (square) and 6 mg/kg (triangle) along with the vehicle control (circle). Tumor samples were analyzed for changes in mRNA levels of EMT markers (**E**) *VIM*, (**F**) *N-CAD*, (**G**) *TWIST1*, and (**H**) *E-CAD* and cytokines (**I**) *IFN-Β* and (**J**) *CXCL-10* upon treatment. Ct values of markers were normalized with respective Ct values for *GAPDH* and fold change was plotted in comparison with the vehicle control. The *p*-values for all the treated groups were compared with the vehicle control group using ordinary one-way analysis of variance followed by Dunnett’s test. For comparison between two internal groups, Dunnett’s multiple comparison test was performed. * *p* < 0.05, ** *p* < 0.01, *** *p* < 0.001, **** *p* < 0.0001, ns: non-significant.

Given that the efficacy of AVA-NP-695 stabilizes 2′3′-cGAMP levels to activate the STING pathway, leading to cytokine release, we evaluated its potency in vivo to inhibit tumor growth in the 4T1 TNBC syngeneic model, which is known to be immune-checkpoint non-responsive [46]. We determined the anti-tumor efficacy of AVA-NP-695 in comparison to approved drugs such as Olaparib (PARP inhibitor) and anti-PD1. In the 4T1 syngeneic mouse model, the lowest dose of AVA-NP-695 (1 mg/kg, BID, PO) was more effective in reducing the tumor size by 40% (*p* < 0.0001) as compared to anti-PD1 (28%, *p* < 0.0001) and Olaparib (33%, *p* < 0.0001) alone. We noticed a 53% decrease (*p* < 0.0001) in the size of the tumor with the highest dose of AVA-NP-695 (6 mg/kg, BID, PO) (Figure 6A,B). Considering the significant decrease in tumor growth, lung metastasis of 4T1 tumors was also evaluated. Interestingly, significant reductions in the lung metastatic nodules were observed in 6 mg/kg AVA-NP-695 group (mean: 41 nodules) compared to the control group (mean: 70 nodules) (Figure 6C and Figure S8A). Histopathological analysis depicted that at 6 mg/kg, BID of AVA-NP-695 treatment, the lung metastatic area was significantly reduced (*p* < 0.0001) by 77% compared to the vehicle control (Figure 6D and Figure S8B). No significant change in body weight was observed during the study (Figure S8C), suggesting that at the tested doses of AVA-NP-695 were well tolerated. Repeat dose studies in mice wherein the AVA-NP-695 was dosed orally twice daily did not show any adverse events or impact on body weight, suggesting a large therapeutic window for the compound (Figure S8D).

Since it was previously demonstrated that the combined treatment of AVA-NP-695 and 2′3′-cGAMP downregulates the expression of EMT markers in MDA-MB-231, we evaluated the mRNA expression levels of various EMT markers in 4T1 tumor samples from a syngeneic mouse model. Consistent with the in vitro data, mesenchymal markers such as *VIM* (*p* < 0.0026), *N-CAD* (*p* < 0.0002), and *TWIST1* (*p* < 0.0378) were significantly downregulated (fold change of 0.5) with 6 mg/kg BID AVA-NP-695 treatment (Figure 6E–G). Subsequently, the expression of *E-CAD* (*p* < 0.0259) (Figure 6H) was marginally upregulated by 1.5-fold in the AVA-NP-695 6 mg/kg BID arm. This correlates well with the observation in Figure 6C,D, where a significant decrease in the lung metastatic area was documented upon AVA-NP-695 treatment. In addition, *IFN-β* (*p* < 0.0004) and *CXCL-10* (*p* < 0.0002) were also found to be upregulated by 4-fold upon dose-dependent treatment of AVA-NP-695 in the 4T1 tumor (Figure 6I,J). Predominantly, these data indicate that administration of AVA-NP-695 could suppress the mesenchymal markers, thereby blocking metastasis, and also inducing expression of *IFN-β* and CXCL-10 necessary to activate T cell-mediated anti-tumor efficacy, thus exhibiting a potential characteristic of a cancer immunotherapy.

## 4. Discussion

A major interest in cancer immunotherapy is finding ways to render immunologically cold tumors hot by immune activation mechanisms. The importance of the cGAS–STING pathway in this context has been well documented [4,47]. Tumor cell-derived DNA activates the cGAS–STING pathway that induces T cell activation both in vivo and in vitro [48]. Interestingly, tumor cells bypass the excess immune activation via several mechanisms such as increasing checkpoint protein expression, T cell exhaustion, etc. [49,50]. As hypothesized and demonstrated by Li et al., overexpression of *ENPP1* also qualifies as one such immune evasion mechanism to deactivate the 2′3′-cGAMP-mediated STING pathway activation [18]. *ENPP1* expression was found to be significantly high in several cancer patients (TCGA dataset), including in breast cancer primary tissues, which showed the highest *ENPP1* expression [18]. The gene expression profile of patient datasets was found to be in line with the cell line datasets, and upon analysis, we found MDA-MB 231, a metastatic breast cancer cell line, showing the highest *ENPP1* levels and A549, a lung adenocarcinoma cell line, showing the least *ENPP1* expression of the given data. After analyzing *ENPP1* expression from patients with different cancer stages, it was also observed that *ENPP1* expression was associated with cancer progression, with advanced-stage patients having high *ENPP1* levels compared to the early-stage patients (Figure 1). This result is consistent with the previous report by Li et al., where *ENPP1* expression depicted a stepwise increase in the various stages of tumorigenesis in a genetically engineered mouse model of lung adenocarcinoma [18]. Being involved in a crucial immune deactivation process, ENPP1 has been rigorously studied as an immuno-oncological target by various research groups worldwide. Our efforts to develop a potent ENPP1 inhibitor resulted in the development of AVA-NP-695 (IC_50_ of 14 nM for TMP as a substrate, sub-nanomolar for cGAMP as a substrate). AVA-NP-695 showed selectivity towards the ENPP1 isoform and did not inhibit other isoforms (ENPP2, ENPP3, ENPP5, ENPP6, and ENPP7) (Figure 2). Metastasis of cancer is driven by EMT [39], where the epithelial cells lose their epithelial features and acquire mesenchymal characteristics [39]. Apart from the immuno-modulatory effect of 2′3′-cGAMP, a recent study has suggested the role of 2′3′-cGAMP in modulating cancer metastasis by negatively regulating EMT [37]. Hence, we explored the role of ENPP1 in cancer progression. Multiple reports have focused on the role of ENPP1 in metastasis [18,19]; however, the exact molecular mechanism is yet to be explored. Recently, Cheng et al. demonstrated that metastasis in CT26 tumor-bearing mice was inhibited by 2′3′-cGAMP via modulating the EMT process, although the major focus was on the effects of 2′3′-cGAMP regulating CD8^+^ T cells and myeloid-derived suppressor cells (MDSCs) in the tumor microenvironment [37]. Here, for the first time, we have demonstrated that 2′3′-cGAMP negatively regulates the EMT markers based on the cellular *ENPP1* expression status. Consistent with our bioinformatics analysis, we observed that *ENPP1* levels were significantly higher in MDA-MB 231 cells compared to A549 cells. 2′3′-cGAMP negatively regulated EMT markers *VIM*, *N-CAD*, and *TWIST* in low-ENPP1-expressing A549 cells (Figure 3), while in high-ENPP1-expressing MDA-MB-231 cells, the impact was negligible, thus demonstrating that ENPP1-mediated hydrolysis of 2′3′-cGAMP can abrogate its beneficial effects on metastasis. Moreover, the impact of 2′3′-cGAMP on the A549 cells resulting in increased *IFN-β*, *CXCL-10,* and *IL-6* mRNA expression was evident (Figure 3). Interestingly, combined treatment of AVA-NP-695 treatment and 2′3′-cGAMP treatment effectively reduces metastasis markers in MDA-MB-231 cells. Direct involvement of ENPP1 in this phenomenon was further confirmed by adding recombinant ENPP1 to A549 cells, which reversed the EMT levels back to those without 2′3′-cGAMP treatment (Figure 4). We further observed that TGF-β-induced EMT was restored back to control in the presence of both 2′3′-cGAMP and AVA-NP-695. This suggests that in a given situation where both TGF-β and 2′3′-cGAMP are present, 2′3′-cGAMP is insufficient and requires AVA-NP-695 to restore EMT (Figure 5). Hence, these data delineate an interesting aspect of ENPP1 upregulation as cancer metastasizes, probably to destabilize the endogenous 2′3′-cGAMP, which can negatively regulate EMT via the Wnt/ꞵ-catenin pathway [37]. Hence, it seems likely that the tumor cells balance out the anti-tumor impact of the STING pathway by increasing ENPP1 expression, which also happens to induce metastasis.

**Figure 7:**
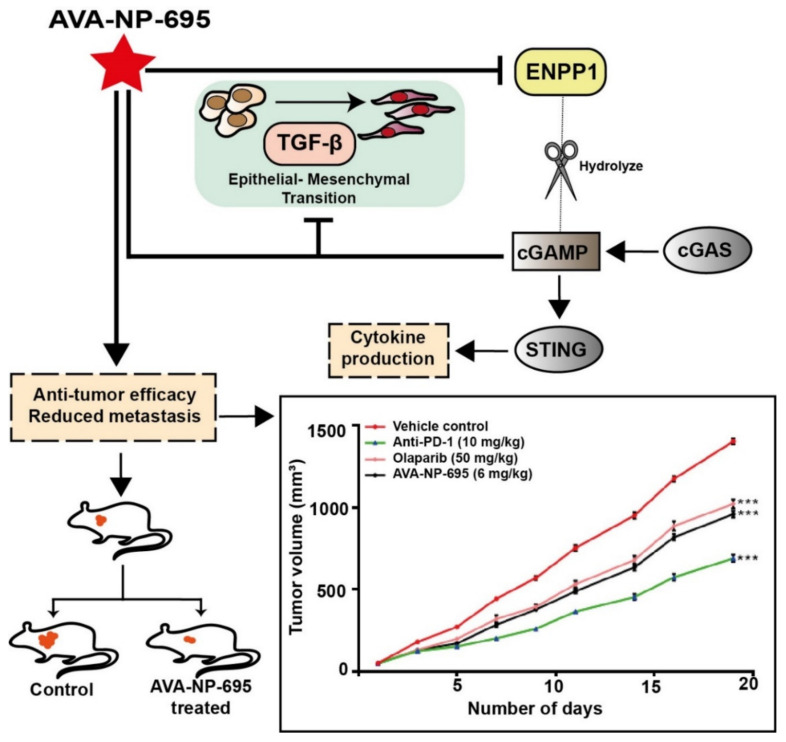
Schematic representation of the study. Apart from immune cell activation, ENPP1 inhibition increases cGAMP levels, thereby negatively regulating the epithelial–mesenchymal transition crucial for metastasis. Hence, ENPP1 inhibition reflects negatively on tumor growth not only by activating immune cells but also by reducing metastasis. *** *p* < 0.001.

The impact of AVA-NP-695 on tumor growth and metastasis was further validated in an in vivo efficacy study using the 4T1 TNBC syngeneic mouse model. Treatment with AVA-NP-695 not only showed higher tumor growth inhibition compared to the existing PARP inhibitor, Olaparib, and anti-PD1, but it also had a comparable impact on reducing the 4T1 lung metastasis (Figure 6). In summary, our data demonstrate the efficacy of ENPP1 inhibitor AVA-NP-695 both in vitro and in vivo. ENPP1 inhibition most likely increases the 2′3′-cGAMP levels, which upregulate the interferon signaling via the STING pathway necessary to activate the T cell-mediated immunity [51,52]. Additionally, it also negatively regulates the epithelial–mesenchymal transition crucial for metastasis (Figure 7).

## Data Availability

The data presented in this study are available in Supplementary Materials.

## Supplementary Materials

The following supporting information can be downloaded at: https://www.mdpi.com/article/10.3390/molecules27196721/s1, Figure S1: The gene expression of ENPP1 in TCGA dataset across breast cancer stages; Figure S2: Selectivity of AVA-NP-695 across isoforms and kinases. Figure S3: Dose dependent effect of 2’3’-cGAMP on IFN induction in THP1 Dual™ cells. Figure S4: Effect of cGAMP and ENPP1 inhibitor on E-cadherin and IFN-ꞵ in MDA-MB-231. Figure S5: Effect of 2’3’-cGAMP on EMT markers in low ENPP1 cells (A549) is mediated by STING. Figure S6: TGF-β treatment in MDA-MB-231 cells. Figure S7: Representative image to show the effect of 2’3’-cGAMP and TGF-ꞵ in presence of AVA-NP-695 on the cell elongation of MDA MB-231 cells. Figure S8: Representative lung metastatic nodules and histopathology image with respect to Figure 6C; Table S1: Raw data for kinome panel. Table S2: Individual cell length post-treatment at 24 h, 48 h and 72 h (related to figure 5 A-D). Table S3: Primer sequence list.

## References

[1] Bray F., Laversanne M., Weiderpass E., Soerjomataram I. (2021). The ever-increasing importance of cancer as a leading cause of premature death worldwide. Cancer.

[2] Zhang Y., Zhang Z. (2020). The history and advances in cancer immunotherapy: Understanding the characteristics of tumor-infiltrating immune cells and their therapeutic implications. Cell. Mol. Immunol..

[3] Seidel J.A., Otsuka A., Kabashima K. (2018). Anti-PD-1 and Anti-CTLA-4 Therapies in Cancer: Mechanisms of Action, Efficacy, and Limitations. Front. Oncol..

[4] Jiang M., Chen P., Wang L., Li W., Chen B., Liu Y., Wang H., Zhao S., Ye L., He Y. (2020). cGAS-STING, an important pathway in cancer immunotherapy. J. Hematol. Oncol..

[5] Ng K.W., Marshall E.A., Bell J.C., Lam W.L. (2018). cGAS-STING and Cancer: Dichotomous Roles in Tumor Immunity and Development. Trends Immunol..

[6] Pépin G., Gantier M.P., Xu D. (2017). cGAS-STING Activation in the Tumor Microenvironment and its Role in Cancer Immunity. Regulation of Inflammatory Signaling in Health and Disease.

[7] Baguley B.C., Ching L.M. (1997). Immunomodulatory actions of xanthenone anticancer agents. BioDrugs Clin. Immunother. Biopharm. Gene Ther..

[8] Roberts Z.J., Goutagny N., Perera P.Y., Kato H., Kumar H., Kawai T., Akira S., Savan R., van Echo D., Fitzgerald K.A. (2007). The chemotherapeutic agent DMXAA potently and specifically activates the TBK1-IRF-3 signaling axis. J. Exp. Med..

[9] Carozza J.A., Böhnert V., Nguyen K.C., Skariah G., Shaw K.E., Brown J.A., Rafat M., von Eyben R., Graves E.E., Glenn J.S. (2020). Extracellular cGAMP is a cancer-cell-produced immunotransmitter involved in radiation-induced anticancer immunity. Nat. Cancer.

[10] Zhou Y., Fei M., Zhang G., Liang W.C., Lin W., Wu Y., Piskol R., Ridgway J., McNamara E., Huang H. (2020). Blockade of the Phagocytic Receptor MerTK on Tumor-Associated Macrophages Enhances P2X7R-Dependent STING Activation by Tumor-Derived cGAMP. Immunity.

[11] Hoong B.Y.D., Gan Y.H., Liu H., Chen E.S. (2020). cGAS-STING pathway in oncogenesis and cancer therapeutics. Oncotarget.

[12] Motedayen Aval L., Pease J.E., Sharma R., Pinato D.J. (2020). Challenges and Opportunities in the Clinical Development of STING Agonists for Cancer Immunotherapy. J. Clin. Med..

[13] Harrington K.J., Brody J., Ingham M., Strauss J., Cemerski S., Wang M., Tse A.N., Khilnani A.D., Marabelle A., Golan T. (2018). Preliminary results of the first-in-human (FIH) study of MK-1454, an agonist of stimulator of interferon genes (STING), as monotherapy or in combination with pembrolizumab (pembro) in patients with advanced solid tumors or lymphomas. Ann. Oncol. Off. J. Eur. Soc. Med. Oncol..

[14] Onyedibe K.I., Wang M., Sintim H.O. (2019). ENPP1, an Old Enzyme with New Functions, and Small Molecule Inhibitors-A STING in the Tale of ENPP1. Molecules.

[15] Li L., Yin Q., Kuss P., Maliga Z., Millán J.L., Wu H., Mitchison T.J. (2014). Hydrolysis of 2’3’-cGAMP by ENPP1 and design of nonhydrolyzable analogs. Nat. Chem. Biol..

[16] Kawaguchi M., Han X., Hisada T., Nishikawa S., Kano K., Ieda N., Aoki J., Toyama T., Nakagawa H. (2019). Development of an ENPP1 Fluorescence Probe for Inhibitor Screening, Cellular Imaging, and Prognostic Assessment of Malignant Breast Cancer. J. Med. Chem..

[17] Pérez-Valencia J.A., Prosdocimi F., Cesari I.M., da Costa I.R., Furtado C., Agostini M., Rumjanek F.D. (2018). Angiogenesis and evading immune destruction are the main related transcriptomic characteristics to the invasive process of oral tongue cancer. Sci. Rep..

[18] Li J., Duran M.A., Dhanota N., Chatila W.K., Bettigole S.E., Kwon J., Sriram R.K., Humphries M.P. (2021). Metastasis and Immune Evasion from Extracellular cGAMP Hydrolysis, a Therapeutic Target in Chromosomally Unstable Tumors. Cancer Discov..

[19] Lau W.M., Doucet M., Stadel R., Huang D., Weber K.L., Kominsky S.L. (2013). Enpp1: A potential facilitator of breast cancer bone metastasis. PLoS ONE.

[20] Wang J., Lu S.F., Wan B., Ming S.L., Li G.L., Su B.Q., Liu J.Y., Wei Y.S., Yang G.Y., Chu B.B. (2018). Maintenance of cyclic GMP-AMP homeostasis by ENPP1 is involved in pseudorabies virus infection. Mol. Immunol..

[21] Sautter C.A., Auray G., Python S., Liniger M., Summerfield A. (2018). Phenotypic and functional modulations of porcine macrophages by interferons and interleukin-4. Dev. Comp. Immunol..

[22] Vijayan D., Young A., Teng M.W.L., Smyth M.J. (2017). Targeting immunosuppressive adenosine in cancer. Nat. Rev. Cancer.

[23] Mittal V. (2018). Epithelial Mesenchymal Transition in Tumor Metastasis. Annu. Rev. Pathol..

[24] Heerboth S., Housman G., Leary M., Longacre M., Byler S., Lapinska K., Willbanks A., Sarkar S. (2015). EMT and tumor metastasis. Clin. Transl. Med..

[25] Eliahu S., Lecka J., Reiser G., Haas M., Bigonnesse F., Levesque S.A., Pelletier J., Sevigny J., Fischer B. (2010). Diadenosine 5’,5’’-(boranated)polyphosphonate analogues as selective nucleotide pyrophosphatase/phosphodiesterase inhibitors. J. Med. Chem..

[26] Nadel Y., Lecka J., Gilad Y., Ben-David G., Forster D., Reiser G., Kenigsberg S., Camden J., Weisman G.A., Senderowitz H. (2014). Highly potent and selective ectonucleotide pyrophosphatase/phosphodiesterase I inhibitors based on an adenosine 5’-(alpha or gamma)-thio-(alpha, beta- or beta, gamma)-methylenetriphosphate scaffold. J. Med. Chem..

[27] Forcellini E., Boutin S., Lefebvre C.-A., Shayhidin E.E., Boulanger M.-C., Rhéaume G., Barbeau X., Lagüe P., Mathieu P., Paquin J.-F. (2018). Synthesis and biological evaluation of novel quinazoline-4-piperidinesulfamide derivatives as inhibitors of NPP1. Eur. J. Med. Chem..

[28] Carozza J.A., Brown J.A., Böhnert V., Fernandez D., AlSaif Y., Mardjuki R.E., Smith M., Li L. (2020). Structure-Aided Development of Small-Molecule Inhibitors of ENPP1, the Extracellular Phosphodiesterase of the Immunotransmitter cGAMP. Cell Chem. Biol..

[29] Gangar M., Goyal S., Raykar D., Khurana P., Martis A.M., Goswami A., Ghoshal I., Patel K.V., Nagare Y., Raikar S. (2022). Design, synthesis and biological evaluation studies of novel small molecule ENPP1 inhibitors for cancer immunotherapy. Bioorganic Chem..

[30] Chandrashekar D.S., Bashel B., Balasubramanya S.A.H., Creighton C.J., Ponce-Rodriguez I., Chakravarthi B., Varambally S. (2017). UALCAN: A Portal for Facilitating Tumor Subgroup Gene Expression and Survival Analyses. Neoplasia.

[31] Cerami E., Gao J., Dogrusoz U., Gross B.E., Sumer S.O., Aksoy B.A., Jacobsen A., Byrne C.J., Heuer M.L., Larsson E. (2012). The cBio cancer genomics portal: An open platform for exploring multidimensional cancer genomics data. Cancer Discov..

[32] Gao J., Aksoy B.A., Dogrusoz U., Dresdner G., Gross B., Sumer S.O., Sun Y., Jacobsen A., Sinha R., Larsson E. (2013). Integrative Analysis of Complex Cancer Genomics and Clinical Profiles Using the cBioPortal. Sci. Signal..

[33] Wang H., Ye F., Zhou C., Cheng Q., Chen H. (2021). High expression of ENPP1 in high-grade serous ovarian carcinoma predicts poor prognosis and as a molecular therapy target. PLoS ONE.

[34] Hu M., Guo W., Liao Y., Xu D., Sun B., Song H., Wang T., Kuang Y., Jing B., Li K. (2019). Dysregulated ENPP1 increases the malignancy of human lung cancer by inducing epithelial-mesenchymal transition phenotypes and stem cell features. Am. J. Cancer Res..

[35] Krug K., Jaehnig E.J., Satpathy S., Blumenberg L., Karpova A., Anurag M., Miles G., Mertins P., Geffen Y., Tang L.C. (2020). Proteogenomic Landscape of Breast Cancer Tumorigenesis and Targeted Therapy. Cell.

[36] Kato K., Nishimasu H., Okudaira S., Mihara E., Ishitani R., Takagi J., Aoki J., Nureki O. (2012). Crystal structure of Enpp1, an extracellular glycoprotein involved in bone mineralization and insulin signaling. Proc. Natl. Acad. Sci. USA.

[37] Cheng H., Xu Q., Lu X., Yuan H., Li T., Zhang Y., Tan X. (2020). Activation of STING by cGAMP Regulates MDSCs to Suppress Tumor Metastasis via Reversing Epithelial-Mesenchymal Transition. Front. Oncol..

[38] Haag S.M., Gulen M.F., Reymond L., Gibelin A., Abrami L., Decout A., Heymann M., van der Goot F.G., Turcatti G., Behrendt R. (2018). Targeting STING with covalent small-molecule inhibitors. Nature.

[39] Ribatti D., Tamma R., Annese T. (2020). Epithelial-Mesenchymal Transition in Cancer: A Historical Overview. Transl. Oncol..

[40] Kim B.N., Ahn D.H., Kang N., Yeo C.D., Kim Y.K., Lee K.Y., Kim T.-J., Lee S.H., Park M.S., Yim H.W. (2020). TGF-β induced EMT and stemness characteristics are associated with epigenetic regulation in lung cancer. Sci. Rep..

[41] Edlund S., Landström M., Heldin C.H., Aspenström P. (2002). Transforming growth factor-beta-induced mobilization of actin cytoskeleton requires signaling by small GTPases Cdc42 and RhoA. Mol. Biol. Cell.

[42] Hao Y., Baker D., ten Dijke P. (2019). TGF-β-Mediated Epithelial-Mesenchymal Transition and Cancer Metastasis. Int. J. Mol. Sci..

[43] O’Connor J.W., Gomez E.W. (2013). Cell Adhesion and Shape Regulate TGF-Beta1-Induced Epithelial-Myofibroblast Transition via MRTF-A Signaling. PLoS ONE.

[44] Wang H., Guo S., Kim S.-J., Shao F., Ho J.W.K., Wong K.U., Miao Z., Hao D., Zhao M., Xu J. (2021). Cisplatin prevents breast cancer metastasis through blocking early EMT and retards cancer growth together with paclitaxel. Theranostics.

[45] Jalalirad M., Haddad T.C., Salisbury J.L., Radisky D., Zhang M., Schroeder M., Tuma A., Leof E., Carter J.M., Degnim A.C. (2021). Aurora-A kinase oncogenic signaling mediates TGF-β-induced triple-negative breast cancer plasticity and chemoresistance. Oncogene.

[46] Kim K., Skora A.D., Li Z., Liu Q., Tam A.J., Blosser R.L., Diaz L.A., Papadopoulos N., Kinzler K.W., Vogelstein B. (2014). Eradication of metastatic mouse cancers resistant to immune checkpoint blockade by suppression of myeloid-derived cells. Proc. Natl. Acad. Sci. United States Am..

[47] Yum S., Li M., Frankel A.E., Chen Z.J. (2019). Roles of the cGAS-STING Pathway in Cancer Immunosurveillance and Immunotherapy. Annu. Rev. Cancer Biol..

[48] Woo S.R., Fuertes M.B., Corrales L., Spranger S., Furdyna M.J., Leung M.Y., Duggan R., Wang Y., Barber G.N., Fitzgerald K.A. (2014). STING-dependent cytosolic DNA sensing mediates innate immune recognition of immunogenic tumors. Immunity.

[49] Reiss K.A., Forde P.M., Brahmer J.R. (2014). Harnessing the power of the immune system via blockade of PD-1 and PD-L1: A promising new anticancer strategy. Immunotherapy.

[50] Jiang Y., Li Y., Zhu B. (2015). T-cell exhaustion in the tumor microenvironment. Cell Death Dis..

[51] Larkin B., Ilyukha V., Sorokin M., Buzdin A., Vannier E., Poltorak A. (2017). Cutting Edge: Activation of STING in T Cells Induces Type I IFN Responses and Cell Death. J. Immunol..

[52] Decout A., Katz J.D., Venkatraman S., Ablasser A. (2021). The cGAS–STING pathway as a therapeutic target in inflammatory diseases. Nat. Rev. Immunol..

