# Supplementary material for: AVA-NP-695 Selectively Inhibits ENPP1 to Activate STING Pathway and Abrogate Tumor Metastasis in 4T1 Breast Cancer Syngeneic Mouse Model
Source: Molecules. 2022 Oct 9;27(19):6721. doi: 10.3390/molecules27196721 (PMC9573294; doi:10.3390/molecules27196721)
Supplement: Supplementary file 1 [file molecules-27-06721-s001.zip › Supplementary file_Revised.pdf]

AVA-NP-695 Selectively Inhibits ENPP1 to Activate STING Pathway and Abrogate Tumor Metastasis in 4T1 Breast Cancer Syngeneic Mouse Model

Avijit Goswami[a], Barnali Deb[a], Sandeep Goyal[a], Abhishek Gosavi[a], Mukund Mali[a], Ashwita M. Martis[a], Princy khurana[a], Mukesh Gangar[a], Digambar Raykar[a], Ankita Mohanty[a], Aditya Kulkarni[a,b#]

[a]Aten Porus Lifesciences, Bangalore 560068, India

[b]Avammune Therapeutics, 39, East Lane, Levittown, PA 19054, USA

[#]Corresponding Author

Supplementary Figures

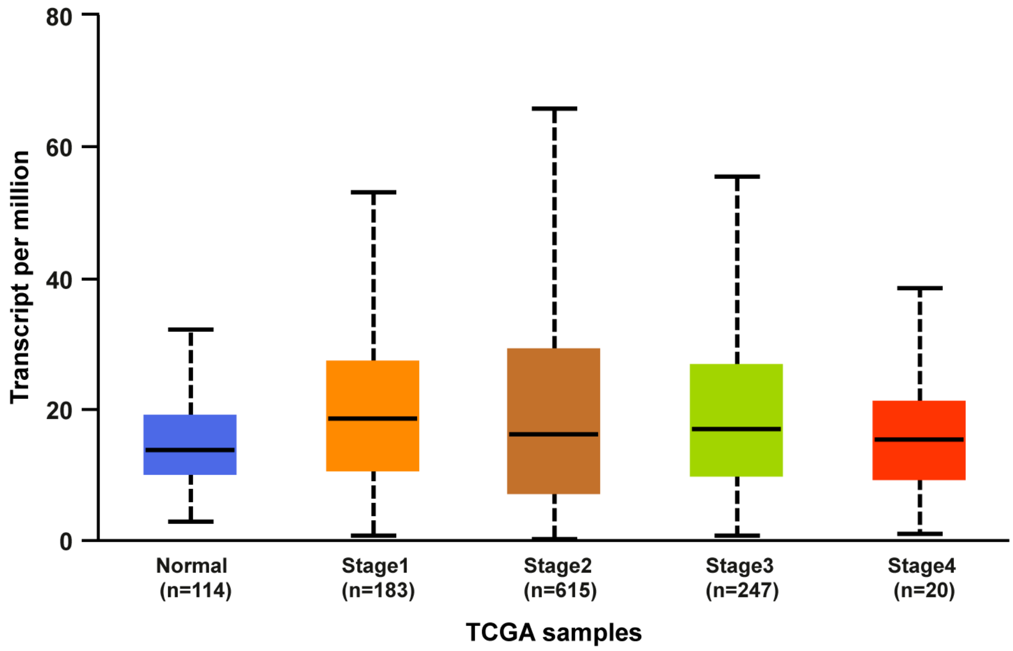

Figure S1: The gene expression of *ENPP1* in TCGA dataset across breast cancer stages. The mRNA levels (transcript per million) of *ENPP1* were plotted for different stages of breast cancer patients from the TCGA dataset (tumor tissue samples). The data analysis was carried out using the UALCAN analysis tool (http://ualcan.path.uab.edu/index.html).

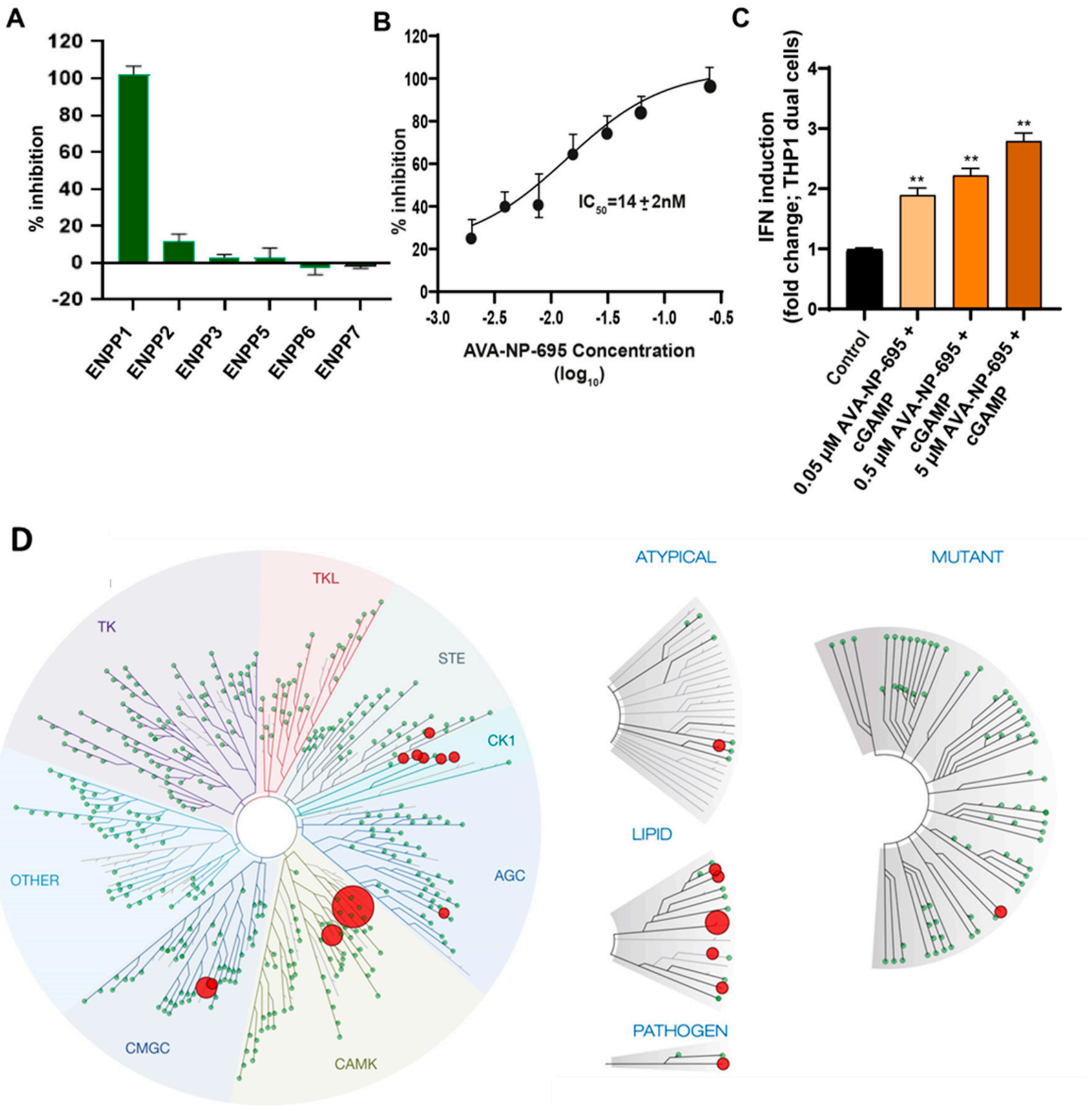

Figure S2: Selectivity of AVA-NP-695 across isoforms and kinases. (**A**) Selectivity assay for ENPP isoforms (NPP2, NPP5, NPP6 and NPP7) (**B**) Dose dependent inhibitory curve. 20 ng of ENPP1 was incubated with increasing concentration of AVA-NP-695 at 37°C followed by addition of 400 µM *p*-Nph-5′-TMP, incubated again at 37°C and reaction was terminated using 1 N NaOH. **(C)** THP1 Dual™ cells were treated with various concentration of AVA-NP-695 for 24h and luciferase assay was performed to check for IFN induction. All the groups were treated with 25 µM of 2′3′-cGAMP and the relative fold change was plotted using control group. **(D)** Selectivity of AVA-NP-695 was tested against 469 kinases at 10 µM concentration using KINOMEscan™ screening platform and image generated using TREE*spot*™ Software Tool and reprinted with permission from KINOME*scan*®, a division of DiscoveRx Corporation, © DISCOVERX CORPORATION 2010.

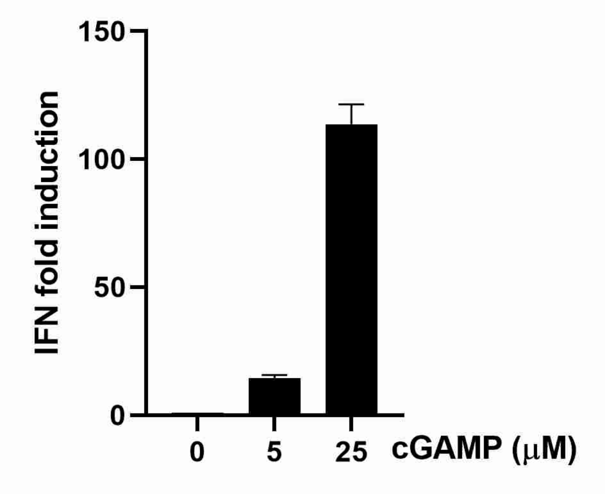

Figure S3: Dose dependent effect of 2'3'-cGAMP on IFN induction in THP1 Dual™ cells. THP1 Dual™ cells were treated with 5μM and 25 μM of 2'3'-cGAMP for 24 h; and the IFN induction was measured using QUANTI-Luc™ luciferase assay.

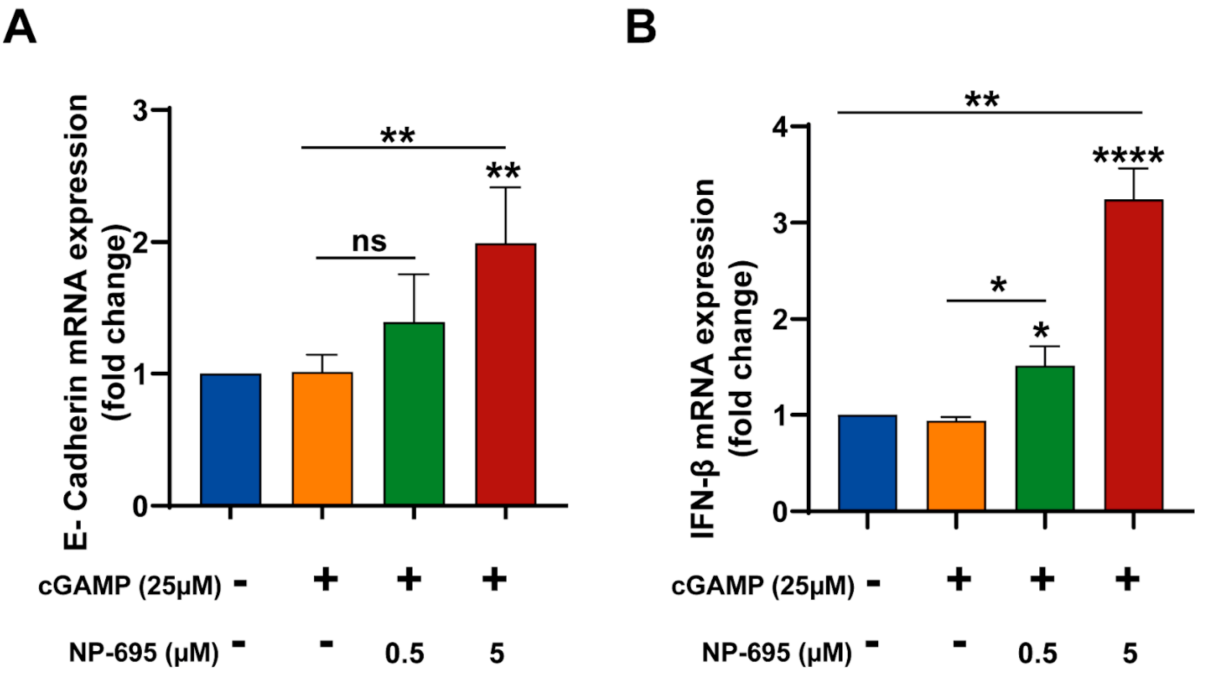

Figure S4: Effect of cGAMP and ENPP1 inhibitor on E-cadherin and IFN-*β* in MDA-MB-231. (A-B) MDA-MB 231 cells were treated with 2'3'-cGAMP (25 μM) in presence of 2 different concentration of ENPP1 inhibitor (0.5 μM and 5 μM); and the mRNA expression level of **(A)** *E-cad* **(B)** *IFN-β* was evaluated through Real Time PCR and data was normalized using GAPDH. **p<0.05, **p<0.01, ***p<0.001, ****p<0.0001.*

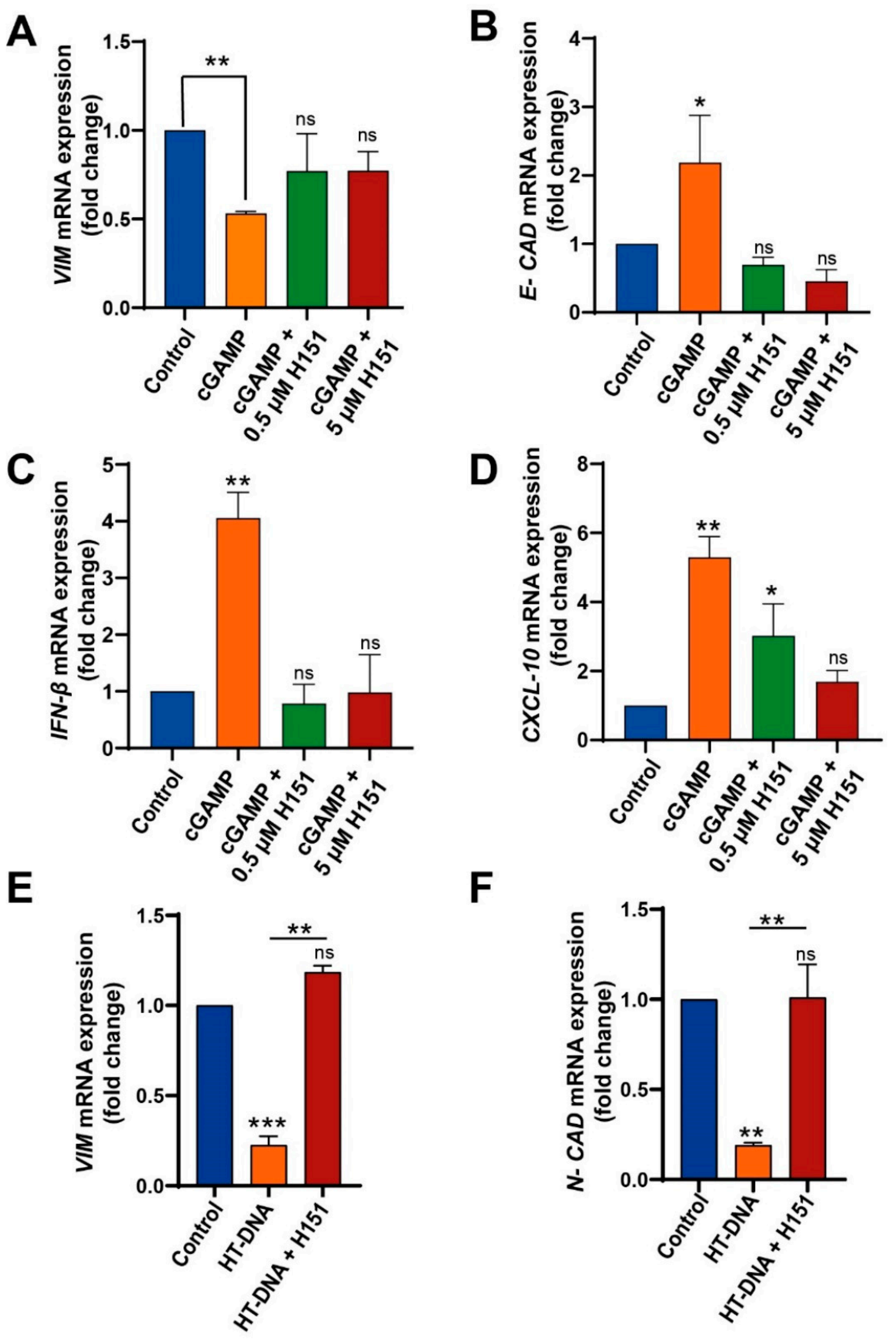

Figure S5: Effect of 2'3'-cGAMP on EMT markers in low ENPP1 cells (A549) is mediated by STING. A549 Dual™ cells were treated with STING inhibitor (H151) in combination with 2'3'-cGAMP (25 µM) and **(A)** *VIM* **(B)** *E-CAD* **(C)** *IFN-β* and **(D)** *CXCL-10* were quantified using Real Time PCR. All the data were normalized using GAPDH. **(E-F)** A549 Dual™ cells were treated with and without 5 µM of H151 for 1 h followed by transfection with 0.5 µg of HT-DNA. Control group were left untreated. Post 24 h treatment, *VIM* **(E)** and *E-CAD* **(F)** mRNA levels were quantified using RT-PCR

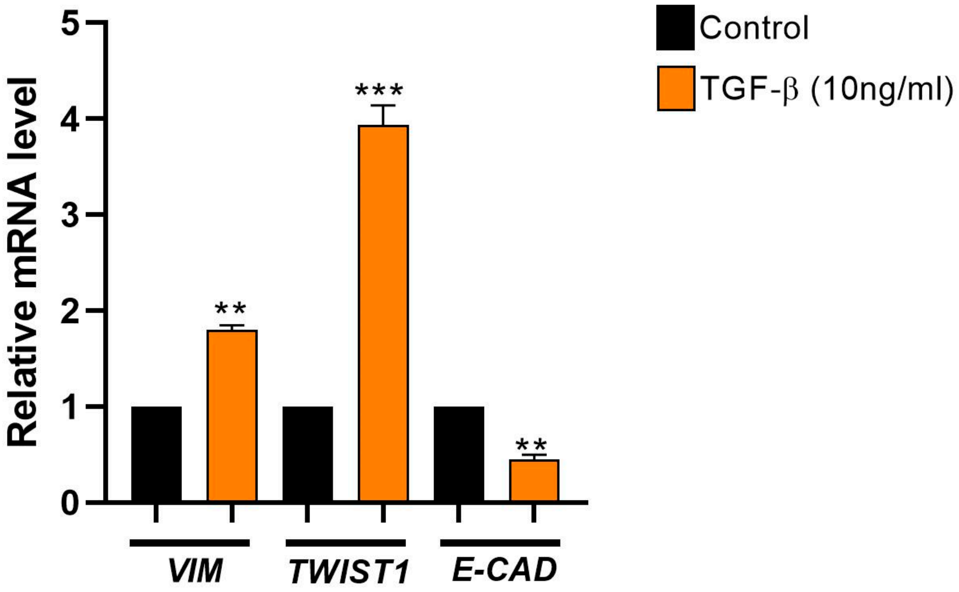

Figure S6: TGF-*β* treatment in MDA-MB-231 cells. MDA-MB-231 cells were treated with 10 ng/ml of TGF-β and *VIM, TWIST1* and *E-CAD* levels were estimated using RT-PCR. All data were normalized using GAPDH as endogenous control.

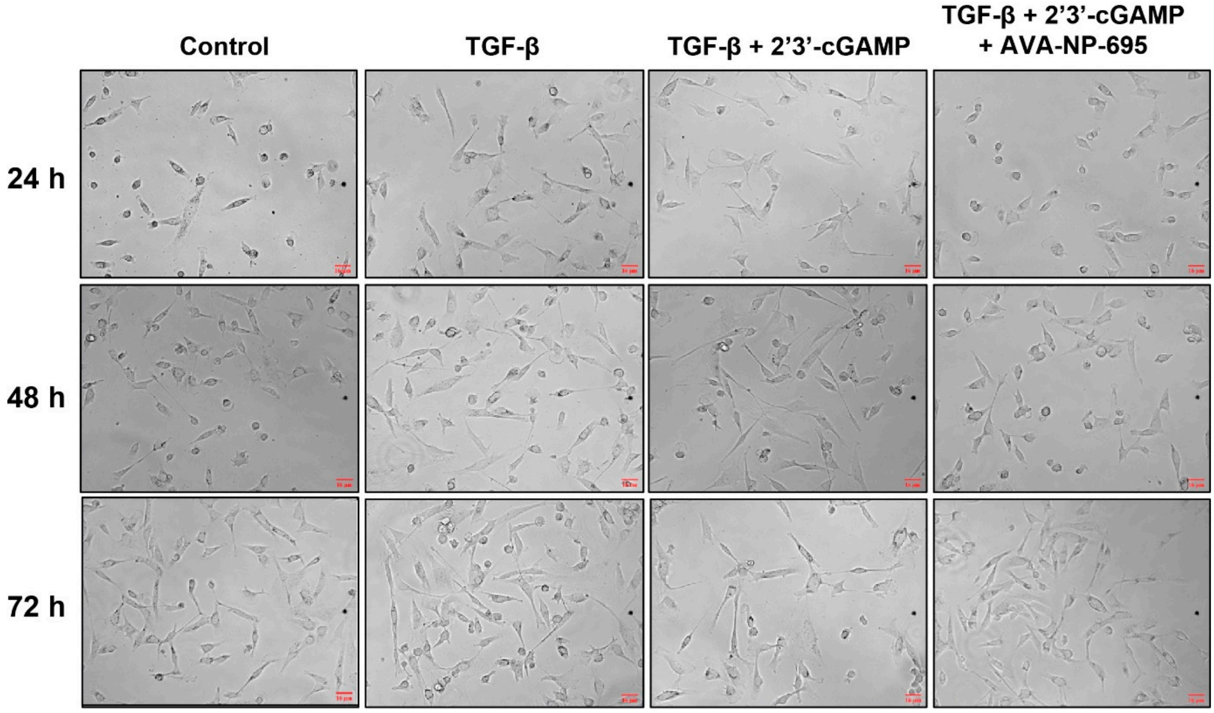

Figure S7: Representative image to show the effect of 2'3'-cGAMP and TGF-β in presence of AVA-NP-695 on the cell elongation of MDA MB-231 cells. The TNBC cell line MDA-MB-231 were treated with different condition and phase contrast image were captured using Floid microscope to quantify the changes in cell length (representative image with respect to Fig. 5).

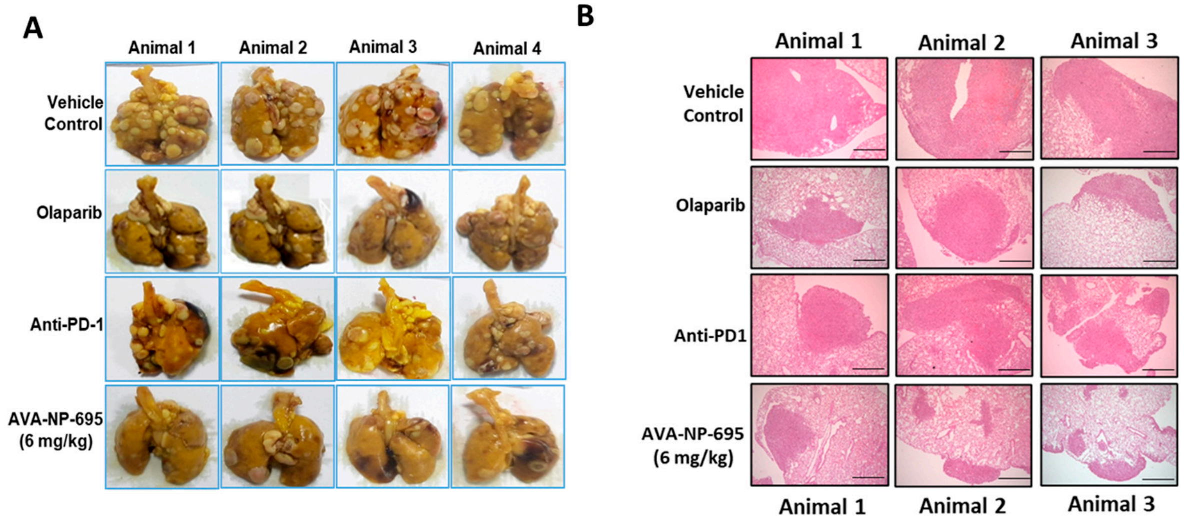

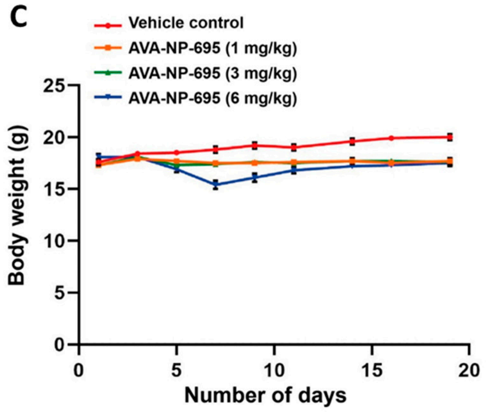

D

Body weight (gm)					Mean		SD		Change in body wt. %
Groups	Treatment	Animal number	Day-0	Day-8	Day-0	Day-8	Day-0	Day-8	Change in body wt. %
G-I	30 mg/kg	1	19.15	19.46	19.07	19.32	0.25	0.30	1.59
		2	18.79	18.98					1.00
		3	19.27	19.52					1.28
G-II	100 mg/kg	4	19.25	19.79	18.99	19.35	0.51	0.59	2.73
		5	19.32	19.59					1.38
		6	18.4	18.68					1.50
G-III	300 mg/kg	7	19.55	19.85	19.75	19.98	0.17	0.14	1.51
		8	19.84	20.13					1.44
		9	19.86	19.96					0.50
G-IV	1000 mg/kg	10	19.85	20.27	19.60	19.89	0.22	0.33	2.07
		11	19.53	19.68					0.76
		12	19.42	19.72					1.52

Figure S8: Representative lung metastatic nodules and histopathology image with respect to Fig. 6 C (A), Fig. 6 D **(B)** and body weights graphs **(C)** for different treatment arms**.** Scale bar corresponds to 4000 µm**. (D)** Female BALB/C mice were treated with different concentration of AVA-NP-695 for 7 days and the body weight was documented at Day 0 and Day 8.

Supplementary Tables

Supplementary Table S2: Individual cell length post-treatment at 24 h, 48 h and 72 h (related to figure 5 A-D)

24h

Control	TGF-β	TGF-β + cGAMP	TGF-β + cGAMP + AVA-NP-695
17.717	51.796	25.087	27.994
19.667	36.634	34.004	34.207
21.606	28.198	28.297	17.022
28.928	47.704	24.654	30.187
17.45	37.558	20.502	24.274
20.25	37.558	21.287	21.057
24.142	31.833	23.322	27.854
14.731	37.999	18.893	41.785
22.669	82.7	31.369	23.91
26.592	59.074	16.229	18.305
10.078	76.255	23.311	27.994
9.939	42.59	35.615	34.207
18.376	34.786	29.303	17.022
16.483	71.65	32.374	30.187
24.012	29.933	48.523	24.274
19.654	32.245	55.442	21.057
23.367	31.43	27.966	27.854
23.57	25.796	31.319	41.785
21.123	35.887	25.125	23.91
27.173	21.287	40.108	18.305
24.271	22.78	17.331	15.321
23.053	43.254	10.809	21.032
23.337	41.023	13.288	23.336
11.607	30.213	15.662	25.254
34.2	22.021	36.25	26.354
13.8	26.554	24.021	40.215
7.862	32.025	22.112	42.031
11.219	51.235	28.556	20.025

48h

Control	TGF-β	TGF-β + cGAMP	TGF-β + cGAMP + AVA-NP-695
20.518	56.869	71.485	27.275
19.952	61.442	42.412	30.153
11.524	58.513	26.117	27.579
25.833	51.808	21.168	22.115
19.588	41.083	24.756	36.24
24.654	36.25	36.726	25.756
23.102	60.244	33.333	20.804
18.276	42.675	32.43	46.056
30.442	51.653	56.452	18.898
38.415	47.704	37.372	31.386
22.81	45.894	57.01	32.599
12.611	41.014	31.953	25.957
24.224	47.909	29.241	20.043
13.044	43.932	40.676	19.194
20.687	37.268	34.593	35.36
28.066	54.88	23.393	32.495
19.76	46.912	57.108	24.051
18.418	42.215	62.121	29.583
19.454	35.785	44.363	35.71
14.919	43.605	35.843	28.29
22.036	41.083	56.153	20.833
30.918	40.32	49.253	13.156
31.128	28.544	45.924	17.5
20.687	57.622	30.187	35.419
27.588	43.908	24.509	27.087
18.028	41.113	24.756	31.669
31.891	51.25	44.653	26.352
24.513	48.376	20.366	36.886
28.52	46.098	34.004	38.193
20.194	47.788	40.494	31.002
18.418	54.038	19.917	26.813
17.936	72.806	60.533	27.415
23.113	29.241	56.312	43.116
10.035	45.417	16.341	33.326
11.785	56.869	20.833	25.087
10.549	61.442	49.211	17.225
16.271	58.513	49.025	14.349
16.796	51.808	77.555	21.81
15.685	41.083	45.966	19.167

11.464	36.25	49.008	26.104
22.989	60.244	47.788	26.014
20.434	42.675	41.164	16.776
19.588	51.653	26.748	26.276
25.583	45.213	23.192	38.263
21.032	50.223	52.879	21.989
32.127	53.235	22.146	36.384
26.874	52.023	17.624	34.291
22.427	56.235	16.713	30.084
21.671	41.021	17.766	23.158
32.449	38.112	21.38	19.508
28.198	32.014	43.016	19.003
24.456	39.322	19.369	29.794
19.117	46.998	27.389	30.259
24.044	45.214	52.15	10.491
12.169	41.236	38.956	16.008
19.742	50.235	29.107	33.046

72 h

Control	TGF-β	TGF-β + cGAMP	TGF-β + cGAMP + AVA-NP-695
27.966	79.005	42.793	26.543
30.76	60.457	49.38	30.382
30.42	43.02	36.539	19.76
32.018	73.245	31.896	20.7
27.528	21.246	48.357	24.615
29.383	44.404	35.129	24.17
30.757	31.647	29.489	28.115
29.516	67.789	42.584	11.524
33.593	61.288	50.118	20.485
25.827	65.418	48.378	11.219
20.017	37.843	34.139	20.833
27.163	36.31	30.052	14.959
26.5	53.865	31.691	38.831
16.796	71.595	50.064	39.528
20.349	40.631	30.233	32.94
18.583	41.516	41.039	30.971
29.63	31.097	43.782	17.525
20.97	28.241	27.265	27.342
28.346	63.45	41.841	15.366
41.384	35.064	45.189	30.462
23.603	57.63	33.917	21.429
35.269	43.161	33.76	28.361

26.306	73.591	37.834	30.075
36.524	45.539	41.777	28.198
26.748	71.318	42.983	21.449
16.029	35.843	65.294	13.649
30.556	51.243	38.006	19.085
26.813	47.041	50.621	20.9
29.089	70.52	42.533	23.143
25.997	61.681	34.646	26.104
23.322	112.067	43.519	20.937
21.767	43.924	40.929	30.348
32.513	40.716	36.764	34.586
35.456	50.09	66.334	11.585
28.336	65.734	48.448	29.533
16.966	41.604	40.539	40.827
31.496	56.864	37.687	16.667
25.611	49.812	94.503	27.945
27.767	107.717	40.322	20.833
26.224	60.625	30.937	33.932
20.022	48.591	49.304	26.874
24.051	30.599	54.823	24.456
17.445	43.231	60.56	34.006
24.559	41.959	41.754	29.557
23.049	50.937	38.364	12.507
23.928	43.167	39.225	18.191
17.678	35.129	45.026	27.163
27.591	107.529	49.998	23.337
28.167	57.185	56.351	36.917
29.098	55.041	61.025	33.863
21.989	37.843	40.256	35.855
31.757	43.995	41.665	24.972
16.008	41.332	49.984	12.388
20.804	65.321	45.213	9.138

Supplementary table S3: Primer sequence list

Gene	Species	Forward Primer	Reverse Primer
**E-Cadherin**	Human	GCCTCCTGAAAAGAGAGTGGAAG	TGGCAGTGTCTCTCCAAATCCG
**N-Cadherin**	Human	CCTCCAGAGTTTACTGCCATGAC	GTAGGATCTCCGCCACTGATTC
**Vimentin**	Human	AGGCAAAGCAGGAGTCCACTGA	ATCTGGCGTTCCAGGGACTCAT
**Twist**	Human	GCCAGGTACATCGACTTCCTCT	TCCATCCTCCAGACCGAGAAGG
**IFN-β**	Human	AGTAGGCGACACTGTTCGTG	GCCTCCCATTCAATTGCCAC
**IL-6**	Human	AGACAGCCACTCACCTCTTCAG	TTCTGCCAGTGCCTCTTTGCTG
**IL-8**	Human	GAGAGAGAGAGAGAGAGAGA	CACA ACC CTC TGC ACC CAG TTT
**CXCL-10**	Human	GGTGAGAAGAGATGTCTGAATCC	GTCCATCCTTGGAAGCACTGCA
**ENPP-1**	Human	CGATTTTGCCGATTGAGGATT	AA CTGGTGCTGGGAAAGAAGACA
**TGF-β**	Human	TACCTGAACCCGTGTTGCTCTC	GTTGCTGAGGTATCGCCAGGAA
**TNF-α**	Human	CTCTTCTGCCTGCTGCACTTTG	ATGGGCTACAGGCTTGTCACTC
**GAPDH**	Human	GTCTCCTCTGACTTCAACAGCG	ACCACCCTGTTGCTGTAGCCAA
**E-Cadherin**	Mouse	GGTCATCAGTGTGCTCACCTCT	GCTGTTGTGCTCAAGCCTTCAC
**N-Cadherin**	Mouse	AGGGTGGACGTCATTGTAGC	CTGTTGGGGTCTGTCAGGAT
**Vimentin**	Mouse	ATGCTTCTCTGGCACGTCTT	AGCCACGCTTTCATACTGCT
**Twist**	Mouse	GGCCGGAGACCTAGATGTCATT	CTGGGAATCTCTGTCCACGG
**CXCL10**	Mouse	ATCATCCCTGCGAGCCTATCCT	GACCTTTTTTGGCTAAACGCTTTC
**IFN-β**	Mouse	GCCTTTGCCATCCAAGAGATGC	ACACTGTCTGCTGGTGGAGTTC
**GAPDH**	Mouse	CATCACTGCCACCCAGAAGACTG	ATGCCAGTGAGCTTCCCGTTCAG
